# Supplementary material for: Insights into the coexistence of birds and humans in cropland through meta-analyses of bird exclosure studies, crop loss mitigation experiments, and social surveys
Source: PLoS Biol. 2023 Jul 6;21(7):e3002166. doi: 10.1371/journal.pbio.3002166 (PMC10325107; doi:10.1371/journal.pbio.3002166)
Supplement: S1 Text — Supporting text for “Search rules.” Table A. Number of crop-consuming bird species by taxonomic order and threatened status. Table B. Eighty-eight crops were included to check whether a species is a crop-feeder or not in the Birds of the World. Table C. Using fail-safe number, Kendall’s rank correlation test, regression test, and “trim and fill” models to test publication bias. The “trim and fill” and regression test does not run for multilevel models in “metafor” package of R (rma.mv), so they were based on random model without multilevel structure (rma). Table D. The significance test of moderators and effect size of subgroups. Q-test was used to test the significance of heterogeneity among comparisons that was attributed to moderators. “Comparisons,” “studies,” and “crops” represent the number of pairwise comparisons, studies, and crop type for each subgroup, respectively. Positive value of effect size means that the presence of birds increases crop production. The lower and upper bounds of 95% confidence interval were reported. Table E. Frequency of landscape variables showing either significant positive, nonsignificant, or significant negative impact of the birds’ effects on crop production. Table F. The significance test of moderators and effect size of subgroups. Averaged effect sizes of multilevel mixed model were calculated for the measures. “Comparisons,” “studies,” and “crops” represent the number of pairwise comparisons, studies, and crop type for each subgroup, respectively. Positive value of effect size means that the measures reduced crop loss caused by birds. The lower and upper bounds of 95% confidence interval were reported. Table G. Models of comparable supports (ΔAICc < 2) for the perceived disservice and positive attitude. Table H. Estimations by the averaged generalized linear mixed-effects binomial models for the disservice. Table I. Estimations by the averaged generalized linear mixed-effects binomial models for the positive attitude. Table J [file pbio.3002166.s007.docx]

**Supplemental Information**

***Insights into the coexistence of birds and humans in cropland through meta-analyses of bird exclosure studies, crop loss mitigation experiments and social surveys***

Cheng Huang, Kaiwen Zhou, Yuanjun Huang, Pengfei Fan, Yang Liu, Tien Ming Lee

**Search rules**

We performed three searches using Web of Science (Web of Science Core Collection, BIOSIS Previews, Chinese Science Citation Database, Derwent Innovation Index, Inspec, KCI-Korean Journal Database, MEDLINE, and SciELO Citation Index) to collect relevant literature in English published during 1950-2020 for the datasets of mitigation, exclosure experiment, and attitude in May, September, and November 2020 by CH (the first author of this study), respectively. We generally used population, intervention, comparator, and outcome (PICO strategies) to define the search rules; we used scientific names of all bird species as search keys in a search rule because some literature did not use the general term "bird" in the title, abstract, and keywords; certain terms were excluded to reduce irrelevant literature. The searches returned articles, reports, conference papers, book sections, and theses in English in the Web of Science. We also snowball-sampled literature cited by relevant papers at the screening stage. We updated the datasets in December 2020 before the final analysis.

In Oct 2022, using the same databases in the Web of Science and search terms as the initial search above, we re-searched non-English literature published before December 2020 and added only one study in Spanish in the exclosure experiment dataset.

For the three datasets, we initially focused on birds and bats; for the mitigation dataset, we initially attempt to evaluate the effectiveness of measures in reducing the loss of crops, fishery, and poultry caused by birds and bats. While the below search keys drew up a longer and broader list of papers, the final meta-analysis was only focused on birds and crops.

**Rules for exclosure experiment dataset**

TS=(bird OR raptor OR "*scientific name of all birds*" OR bat)

**And**

TS=(exclud* OR exclosure OR exclusion OR net OR wire OR service OR disservice)

**And**

TS=(crop OR agriculture OR corn OR sunflower OR tuber OR seed OR maize OR wheat OR *nut OR cherry OR berry OR rice OR soybean OR sorghum OR fruit OR orchard OR apple OR vegetable)

**And**

TS=(yield OR output OR product* OR los* OR biomass OR damage)

**Not**

TS=(broiler OR clinical OR muscle OR metabolism OR dairy OR immun* OR cell OR nerve OR genetic OR genomic OR poultry OR egg)

**Rules for mitigation dataset**

TS=(bird OR raptor OR "*scientific name of all birds*" OR bat)

**And**

TS=(conflict OR damag* OR feed OR raid OR consum* OR predat* OR kill* OR prey)

**And**

TS=(crop OR agriculture OR corn OR sunflower OR tuber OR seed OR maize OR wheat OR *nut OR cherry OR berry OR rice OR soybean OR sorghum OR fruit OR orchard OR apple OR vegetable OR fish* OR pet OR livestock OR chick* OR game* OR peasant OR poult* OR grouse OR goat OR cow OR lamb)

**And**

TS=(yield OR output OR product* OR los* OR death OR biomass OR mortality OR stock OR attitude OR perception)

**And**

TS=(protect* OR manag* OR *control OR mitigation OR mitgat* OR repel* OR compensat* OR guard* OR trap OR scar* OR bait* OR cull* OR avers* OR fenc* OR poison* OR shoot*)

**Not**

TS=(broiler OR clinical OR muscle OR metabolism OR dairy OR immun* OR cell OR nerve OR genetic OR genomic OR poultry OR egg)

**Rules for attitude dataset**

TS=(bird OR raptor OR “*scientific name of all birds*" OR bat)

**And**

TS=(farm* OR village* OR suburb OR countryside OR community OR coexist* OR “nature reserve” OR “national park” OR livelihood OR hunter OR public OR crop OR agriculture OR fruit OR orchard OR vegetable OR citizen OR tourist OR visitor OR urban OR city)

**And**

TS=(attitude OR perception OR awareness OR belief)

**Table A. Number of crop-consuming bird species by taxonomic order and threatened status.**

| Taxonomic order | Not threatened | | Threatened | | | Total |
| --- | --- | --- | --- | --- | --- | --- |
|  | Least Concern | Near Threatened | Vulnerable | Endangered | Critically Endangered |  |
| Passeriformes | 535 | 16 | 24 | 9 | 5 | 589 |
| Psittaciformes | 77 | 17 | 19 | 9 | 6 | 128 |
| Piciformes | 63 | 3 | 3 | 0 | 1 | 70 |
| Columbiformes | 46 | 3 | 4 | 4 | 3 | 60 |
| Anseriformes | 46 | 3 | 4 | 3 | 1 | 57 |
| Galliformes | 39 | 5 | 6 | 3 | 2 | 55 |
| Gruiformes | 17 | 2 | 3 | 1 | 1 | 24 |
| Charadriiformes | 19 | 1 | 1 | 0 | 1 | 22 |
| Bucerotiformes | 5 | 0 | 2 | 0 | 0 | 7 |
| Accipitriformes | 4 | 0 | 0 | 0 | 1 | 5 |
| Struthioniformes | 5 | 0 | 0 | 0 | 0 | 5 |
| Caprimulgiformes | 2 | 1 | 0 | 0 | 1 | 4 |
| Coraciiformes | 4 | 0 | 0 | 0 | 0 | 4 |
| Otidiformes | 0 | 2 | 2 | 0 | 0 | 4 |
| Trogoniformes | 4 | 0 | 0 | 0 | 0 | 4 |
| Coliiformes | 3 | 0 | 0 | 0 | 0 | 3 |
| Musophagiformes | 3 | 0 | 0 | 0 | 0 | 3 |
| Pterocliformes | 3 | 0 | 0 | 0 | 0 | 3 |
| Cathartiformes | 2 | 0 | 0 | 0 | 0 | 2 |
| Cuculiformes | 1 | 0 | 1 | 0 | 0 | 2 |
| Falconiformes | 2 | 0 | 0 | 0 | 0 | 2 |
| Pelecaniformes | 1 | 1 | 0 | 0 | 0 | 2 |
| Cariamiformes | 1 | 0 | 0 | 0 | 0 | 1 |
| Strigiformes | 1 | 0 | 0 | 0 | 0 | 1 |

**Table B. Eighty-eight crops were included to check whether a species is a crop-feeder or not in the Birds of the World.**

| Stem type | Crop | Total |
| --- | --- | --- |
| Herbaceous crops | Barley, Buckwheat, Canary Seed, Fonio, Maize, Millet, Oat, Quinoa, Rice, Rye, Sorghum, Triticale, Wheat, Banana, Pineapple, Plantain, Strawberry, Castor, Groundnut, Hempseed, Linseed, Mustard, Poppy, Rapeseed, Safflower, Sesame, Soybean, Sunflower, Chicory, Pepper, Artichoke, Asparagus, Cabbage, Carrot, Cauliflower, Chilli, Cucumber, Eggplant, Garlic, Lettuce, Okra, Onion, Pea, Pumpkin, Spinach, Tomato, Watermelon | 47 |
| Woody crops | Apple, Apricot, Avocado, Blueberry, Carob, Cashew Apple, Cherry, Citrus, Cocoa, Coffee, Cranberry, Currant, Date, Gooseberry, Grape, Grapefruit, Kiwi, Lemon, Lime, Mango, Orange, Papaya, Peach, Pear, Persimmon, Plum, Quince, Raspberry, Tange, Coconut, Karite, Oil palm, Olive, Tung, Almond, Brazil nut, Cashew, Chestnut, Hazelnut, Pistachio, Walnut | 41 |

**Table C. Using fail-safe number, Kendall’s rank correlation test,** **regression test and “trim and fill” models to test publication bias.** The ‘trim and fill’ and regression test does not run for multilevel models in “metafor” package of R (rma.mv), so they were based on random model without multilevel structure (rma).

|  |  |  | Kendall test | | Regression test | Original | | | Trim and fill | | |  |
| --- | --- | --- | --- | --- | --- | --- | --- | --- | --- | --- | --- | --- |
| Model | Comparison | Fail safe number | z | *p* | *p* | Effect size | Lower | Upper | Effect size | Lower | Upper | |
| All exclosure comparisons | 158 | - | 1.47 | 0.14 | 0.01 | 0.06 | -0.19 | 0.32 | 0.16 | 0.02 | 0.29 | |
| Exclosure comparisons on woody crops | 101 | 2250 | 0.43 | 0.67 | 0.24 | 0.46 | 0.23 | 0.70 | 0.31 | 0.20 | 0.42 | |
| Exclosure comparisons on herbaceous crop | 57 | 2291 | 3.21 | 0.00 | < 0.0001 | -0.48 | -0.80 | -0.16 | -0.22 | -0.45 | 0.01 | |
| All mitigation comparisons | 114 | 39250 | -1.79 | 0.07 | < 0.0001 | 1.20 | 0.81 | 1.60 | 1.41 | 1.07 | 1.74 | |
| All-stage comparisons | 93 | 25230 | -1.30 | 0.19 | < 0.0001 | 1.06 | 0.67 | 1.46 | 1.07 | 0.77 | 1.36 | |
| All-stage repellent comparisons | 32 | 390 | -1.85 | 0.06 | < 0.0001 | 0.85 | 0.39 | 1.32 | 0.31 | -0.14 | 0.77 | |
| Sow-stage comparisons | 21 | 1522 | -2.37 | 0.02 | < 0.0001 | 4.59 | 1.91 | 7.27 | 5.00 | 2.72 | 7.27 | |

**Table D.** **The significance test of moderators and effect size of subgroups.** Q-test was used to test the significance of heterogeneity among comparisons that was attributed to moderators. “Comparisons”, “studies”, and “crops” represent the number of pairwise comparisons, studies, and crop type for each subgroup, respectively. Positive value of effect size means that the presence of birds increases crop production. The lower and upper bounds of 95% confidence interval were reported.

| Crop stem | Moderator | Q_m_ | Q_m_*p* | Subgroups | Comparisons | Studies | Crops | Effect size | Lower | Upper | *p* |
| --- | --- | --- | --- | --- | --- | --- | --- | --- | --- | --- | --- |
| Woody crops | Climate | 1.81 | 0.18 | Non-tropical | 52 | 14 | 4 | 0.67 | 0.29 | 1.06 | 0.00 |
|  |  |  |  | Tropical | 49 | 8 | 5 | 0.34 | 0.05 | 0.63 | 0.02 |
|  | Focal animal | 1.12 | 0.29 | Bird | 74 | 18 | 7 | 0.50 | 0.26 | 0.74 | 0.00 |
|  |  |  |  | Bird and bat | 27 | 8 | 5 | 0.35 | 0.06 | 0.64 | 0.02 |
|  | Food type | 0.61 | 0.89 | Fruit | 28 | 6 | 3 | 0.42 | -0.08 | 0.91 | 0.10 |
|  |  |  |  | Oilcrop | 7 | 2 | 1 | 0.22 | -0.74 | 1.18 | 0.65 |
|  |  |  |  | Coffee and cacao | 43 | 12 | 2 | 0.56 | 0.20 | 0.92 | 0.00 |
|  |  |  |  | Treenut | 23 | 2 | 2 | 0.37 | -0.44 | 1.19 | 0.37 |
|  | Crop type | 4.59 | 0.71 | Almond | 19 | 1 | - | -0.02 | -1.19 | 1.14 | 0.97 |
|  |  |  |  | Apple | 26 | 4 | - | 0.66 | 0.06 | 1.27 | 0.03 |
|  |  |  |  | Cacao | 19 | 4 | - | 0.33 | -0.28 | 0.93 | 0.30 |
|  |  |  |  | Coffee | 24 | 8 | - | 0.70 | 0.24 | 1.16 | 0.00 |
|  |  |  |  | Loquat | 1 | 1 | - | 0.07 | -1.22 | 1.37 | 0.91 |
|  |  |  |  | Lychee | 1 | 1 | - | -0.28 | -1.49 | 0.94 | 0.65 |
|  |  |  |  | Macadamia nut | 4 | 0 | - | 0.78 | -0.40 | 1.96 | 0.20 |
|  |  |  |  | Oil palm | 7 | 2 | - | 0.22 | -0.74 | 1.19 | 0.65 |
|  | Use of herbicide | 0.19 | 0.67 | Herbicide | 21 | 6 | 3 | 0.28 | -0.03 | 0.58 | 0.08 |
|  |  |  |  | No herbicide | 22 | 5 | 3 | 0.18 | -0.13 | 0.50 | 0.25 |
|  | Use of insecticide | 0.06 | 0.81 | Insecticide | 40 | 9 | 3 | 0.59 | 0.20 | 0.98 | 0.00 |
|  |  |  |  | No insecticide | 32 | 11 | 5 | 0.53 | 0.17 | 0.90 | 0.00 |
|  | Regional bird richness | 0.03 | 0.86 | Richness | 64 | 11 | 7 | 0.00 | 0.00 | 0.01 | 0.86 |
| Herbaceous crops | Climate | 6.76 | 0.01 | Non-tropical | 49 | 3 | 3 | -0.62 | -0.93 | -0.32 | 0.00 |
|  |  |  |  | Tropical | 8 | 15 | 9 | 0.40 | -0.31 | 1.10 | 0.27 |
|  | Focal animal | 4.47 | 0.11 | Bird | 28 | 13 | 9 | -0.58 | -0.94 | -0.21 | 0.00 |
|  |  |  |  | Bird and bat | 1 | 1 | 1 | 0.87 | -0.42 | 2.17 | 0.19 |
|  |  |  |  | Geese/duck/crane | 28 | 4 | 3 | -0.48 | -1.06 | 0.10 | 0.10 |
|  | Food type | 10.14 | 0.02 | Cereal | 48 | 12 | 5 | -0.53 | -0.90 | -0.17 | 0.00 |
|  |  |  |  | Fruit | 1 | 1 | 1 | -0.31 | -1.52 | 0.90 | 0.61 |
|  |  |  |  | Oilcrop | 3 | 3 | 2 | -1.37 | -2.03 | -0.71 | 0.00 |
|  |  |  |  | Vegetable | 5 | 3 | 3 | 0.12 | -0.63 | 0.87 | 0.75 |
|  | Crop type | 26.94 | 0.00 | Barley | 4 | 1 | - | 0.07 | -0.59 | 0.73 | 0.84 |
|  |  |  |  | Broccoli | 1 | 1 | - | 0.82 | -0.37 | 2.01 | 0.17 |
|  |  |  |  | Cabbage | 2 | 1 | - | -0.15 | -1.11 | 0.80 | 0.76 |
|  |  |  |  | Kale | 2 | 1 | - | -0.13 | -1.03 | 0.77 | 0.78 |
|  |  |  |  | Maize | 3 | 2 | - | -0.10 | -0.75 | 0.55 | 0.77 |
|  |  |  |  | Millet | 1 | 1 | - | 0.87 | -0.18 | 1.93 | 0.10 |
|  |  |  |  | Rice | 15 | 5 | - | -0.85 | -1.34 | -0.36 | 0.00 |
|  |  |  |  | Soybean | 2 | 2 | - | -0.86 | -1.67 | -0.04 | 0.04 |
|  |  |  |  | Strawberry | 1 | 1 | - | -0.31 | -1.20 | 0.58 | 0.49 |
|  |  |  |  | Sunflower | 1 | 1 | - | -1.79 | -2.89 | -0.68 | 0.00 |
|  |  |  |  | Wheat | 25 | 4 | - | -0.60 | -1.04 | -0.16 | 0.01 |
|  | Use of herbicide | 1.76 | 0.18 | Herbicide | 22 | 3 | 1 | -0.68 | -1.36 | 0.00 | 0.05 |
|  |  |  |  | No herbicide | 7 | 2 | 2 | 0.12 | -0.85 | 1.08 | 0.81 |
|  | Use of insecticide | 0.14 | 0.71 | Insecticide | 4 | 2 | 3 | -0.09 | -0.67 | 0.48 | 0.75 |
|  |  |  |  | No insecticide | 7 | 2 | 2 | 0.08 | -0.62 | 0.77 | 0.83 |
|  | Regional bird richness | 4.49 | 0.03 | Richness | 40 | 11 | 8 | -0.01 | -0.02 | 0.00 | 0.03 |

**Table E. Frequency of landscape variables showing either significant positive, non-significant, or significant negative impact of the birds’ effects on crop production.**

| Variable | Negative | Nonsignificant | Positive |
| --- | --- | --- | --- |
| Distance to edge | 1 | 1 | 0 |
| Distance to primary forest or habitat patch | 0 | 2 | 1 |
| Surrounding forest or habitat coverage | 0 | 26 | 12 |
| Number of banana trees | 0 | 1 | 0 |
| Shade | 2 | 7 | 2 |
| Sum | 3 | 37 | 15 |

**Table F. The significance test of moderators and effect size of subgroups.** Averaged effect sizes of multilevel mixed model were calculated for the measures. “Comparisons”, “studies”, and “crops” represent the number of pairwise comparisons, studies, and crop type for each subgroup, respectively. Positive value of effect size means that the measures reduced crop loss caused by birds. The lower and upper bounds of 95% confidence interval were reported.

| Category | Moderator | Q_m_ | Q_m_*p* | Subgroup | Comparisons | Studies | Crops | Effect size | Lower | Upper | *p* |
| --- | --- | --- | --- | --- | --- | --- | --- | --- | --- | --- | --- |
| All stage | Measure category | 5.55 | 0.48 | Bird perch | 13 | 2 | 2 | 0.31 | -1.34 | 1.95 | 0.71 |
|  |  |  |  | Herbicide to roost | 2 | 1 | 1 | -0.26 | -2.61 | 2.09 | 0.83 |
|  |  |  |  | Net | 6 | 3 | 3 | 1.04 | -0.17 | 2.25 | 0.09 |
|  |  |  |  | Repellent | 32 | 21 | 7 | 0.89 | 0.36 | 1.42 | 0.00 |
|  |  |  |  | Scaring model | 8 | 6 | 6 | 1.23 | 0.35 | 2.12 | 0.01 |
|  |  |  |  | Sound | 11 | 5 | 5 | 0.98 | -0.01 | 1.98 | 0.05 |
|  |  |  |  | Tape/ribbon/flag | 21 | 8 | 8 | 1.76 | 0.97 | 2.54 | 0.00 |
| All stage repellent | Measure category | 10.70 | 0.00 | 4-aminopyridine bait | 8 | 5 | 2 | 0.74 | 0.01 | 1.47 | 0.05 |
|  |  |  |  | Methiocarb | 16 | 13 | 6 | 1.23 | 0.72 | 1.74 | 0.00 |
|  |  |  |  | Methyl anthranilate | 8 | 3 | 3 | -0.45 | -1.32 | 0.42 | 0.31 |
| Sow stage | Measure category | 11.07 | 0.00 | Physical barrier | 2 | 1 | 1 | 7.76 | 1.87 | 13.64 | 0.01 |
|  |  |  |  | Repellent | 10 | 5 | 3 | 1.76 | -0.70 | 4.23 | 0.16 |
|  |  |  |  | Sow practice | 9 | 3 | 3 | 7.94 | 4.98 | 10.90 | 0.00 |

**Table G. Models of comparable supports (ΔAICc < 2) for the perceived disservice and positive attitude.**

| Depedent variable | Indepedent variable | AICc | △AICc | Weight |
| --- | --- | --- | --- | --- |
| Perceived disservice | economic status, bird group | 310.3 | 0.00 | 0.559 |
|  | climate region, economic status, bird group | 311.1 | 0.87 | 0.363 |
| Positive attitude | economic status, bird group | 1815.0 | 0.00 | 0.473 |
|  | bird group | 1816.2 | 1.21 | 0.258 |
|  | climate region, economic status, bird group | 1817.0 | 1.93 | 0.180 |

**Table H.** **Estimations by the averaged generalized linear mixed-effects binomial models for the disservice.**

|  | Perceived disservice | | |
| --- | --- | --- | --- |
| *Predictors* | *Log-Odds* | *CI* | *p* |
| Intercept | -1.06 | -2.25 – 0.13 | 0.080 |
| Low-income | 3.00 | 1.15 – 4.86 | 0.002 |
| Covid | 1.32 | -0.83 – 3.46 | 0.228 |
| Icterid | 3.56 | 1.41 – 5.71 | 0.001 |
| Parrot | 1.35 | -0.80 – 3.49 | 0.218 |
| Wildfowl | 0.12 | -1.50 – 1.75 | 0.881 |
| Tropics | -1.47 | -3.07 – 0.13 | 0.072 |
| N _study_ | 22 | | |
| Observations | 26 | | |

**Table I. Estimations by** **the averaged generalized linear mixed-effects binomial models for the positive attitude.**

|  | Positive attitude | | |
| --- | --- | --- | --- |
| *Predictors* | *Log-Odds* | *CI* | *p* |
| Intercept | 0.92 | -0.34 – 2.18 | 0.152 |
| Low-income | -1.98 | -3.74 – -0.22 | 0.028 |
| Corvid | -1.25 | -2.72 – 0.23 | 0.097 |
| Finch | 0.57 | -0.90 – 2.05 | 0.444 |
| Icterid | 1.28 | -0.25 – 2.81 | 0.102 |
| Other | 0.45 | -1.02 – 1.92 | 0.546 |
| Parrot | 0.22 | -1.25 – 1.69 | 0.771 |
| Pigeon | -0.25 | -1.72 – 1.21 | 0.734 |
| Tit | 2.65 | 1.09 – 4.20 | 0.001 |
| Wildfowl | -1.05 | -2.51 – 0.42 | 0.162 |
| Tropics | 0.92 | -0.54 – 2.39 | 0.218 |
| N _study_ | 22 | | |
| Observations | 50 | | |

**Table J. PICO framework for the exclosure experiment dataset.**

| Question: whether the access of birds to crops affect crop productions? | |
| --- | --- |
| Question key elements | Eligibility criteria |
| Population (P):   - Crop/field excluded from birds | Crops belong to the categories of fruits, treenuts, oilcrops, vegetables, and cereals. Birds are the focal animals. |
| Intervention (I)   - Exclosure | The access of birds to crops was properly manipulated using grids, nets, or frames, which allow the access of arthropods but exclude the birds. |
| Comparator (C)   - Crop/field with free access of birds | Crops belong to the categories of fruits, treenuts, oilcrops, vegetables, and cereals. Birds are the focal animals. |
| Outcome (O)   - Crop/field yield | Full data (mean, sd, and n) or convertible data about crop production were reported, such as the yield per square meter, kg per tree, and number of shoots per square meter. |

**Table K. PICO framework for the mitigating experiment dataset.**

| Question: which measure is effective for reducing crop losses caused by birds? | |
| --- | --- |
| Question key elements | Eligibility criteria |
| Population (P):   - Crop/field with mitigating measures |  |
| Intervention (I)   - Mitigating measures | A single specific measure rather than integrated measures; the measures were targeted to free-ranging wild birds. |
| Comparator (C)   - Crop/field without mitigating measures |  |
| Outcome (O)   - Crop/field yield | Full data (mean, sd, and n) or convertible data about crop production or damage were reported, such as the yield per square meter, kg per tree, and number of shoots per square meter. |

**Table L. Estimations by the averaged generalized linear mixed-effects binomial models for the negative attitude.**

|  | Negative attitude | | |
| --- | --- | --- | --- |
| *Predictors* | *Log-Odds* | *CI* | *p* |
| Intercept | -1.56 | -2.99 – -0.14 | 0.031 |
| Low-income | 2.64 | 0.63 – 4.64 | 0.010 |
| Corvid | 1.32 | -0.29 – 2.93 | 0.108 |
| Finch | -0.48 | -2.09 – 1.13 | 0.556 |
| Icterid | -1.18 | -2.85 – 0.48 | 0.162 |
| Other | -0.38 | -1.99 – 1.23 | 0.644 |
| Parrot | -0.14 | -1.75 – 1.47 | 0.866 |
| Pigeon | 0.35 | -1.26 – 1.96 | 0.669 |
| Tit | -2.57 | -4.25 – -0.88 | 0.003 |
| Wildfowl | 1.16 | -0.45 – 2.76 | 0.157 |
| Tropics | -1.31 | -2.96 – 0.33 | 0.118 |
| N _study_ | 22 | | |
| Observations | 51 | | |

**
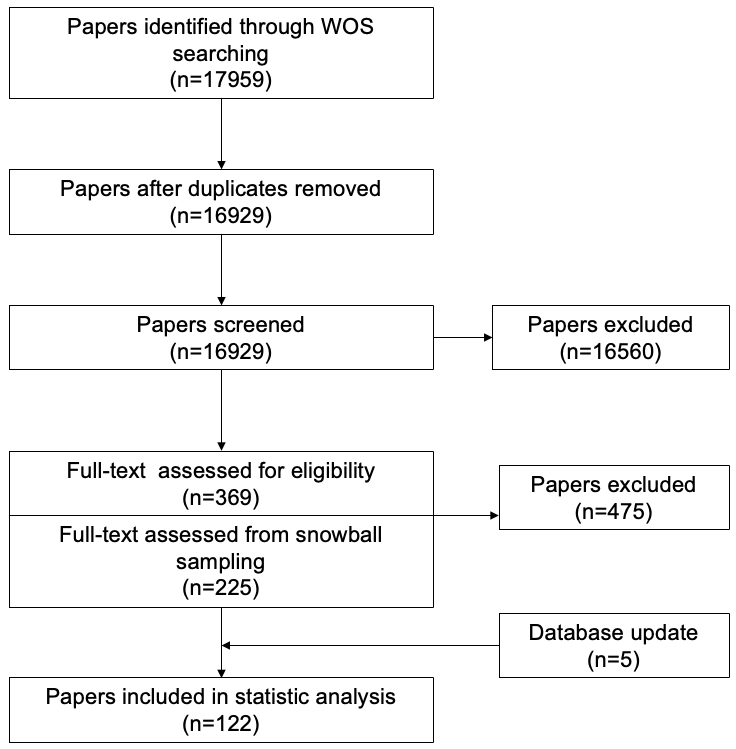
**

**Fig A.** **PRISMA flow of the meta-analyses.**

**
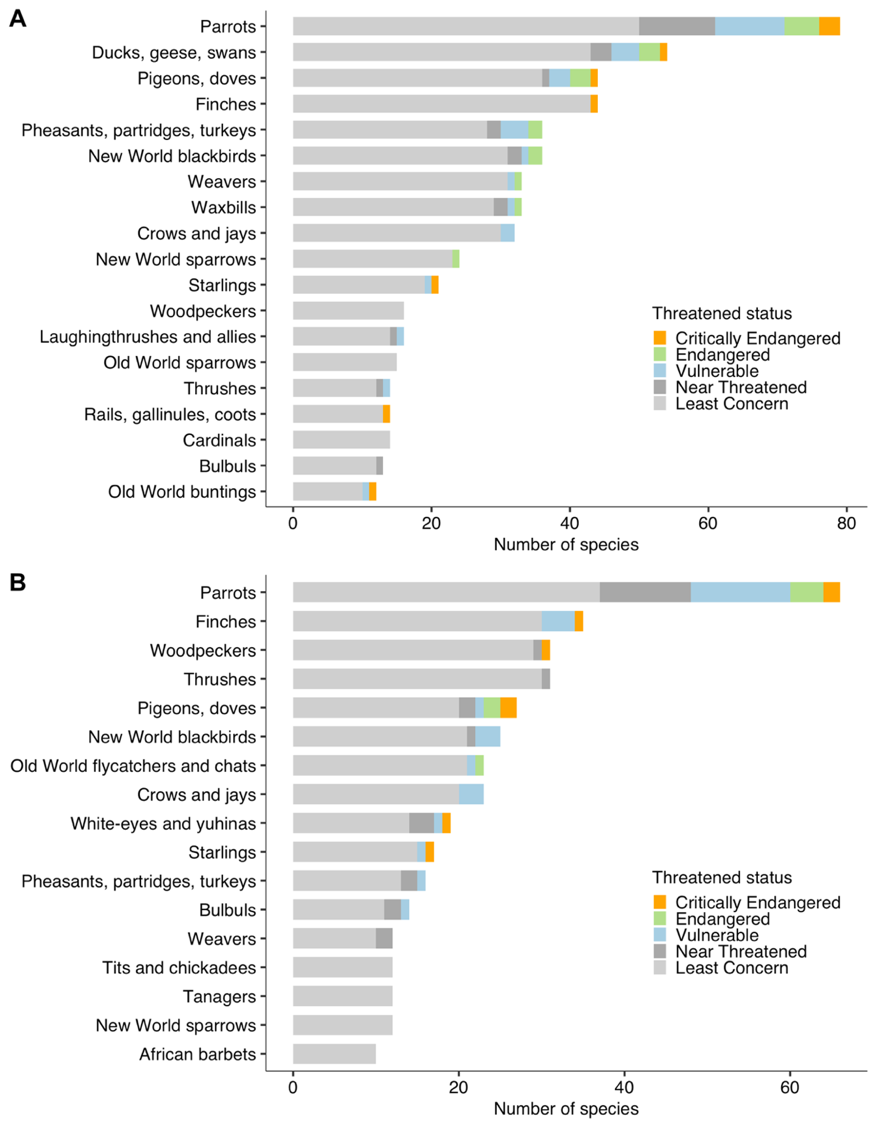
**

**Fig B. Number of crop-consuming species by taxonomic family and threatened status.** The barplots show the major taxon (top 25%) consuming herbaceous (**A**) and woody (**B**) crops. The data underlying this figure can be found in S1 Data.

**
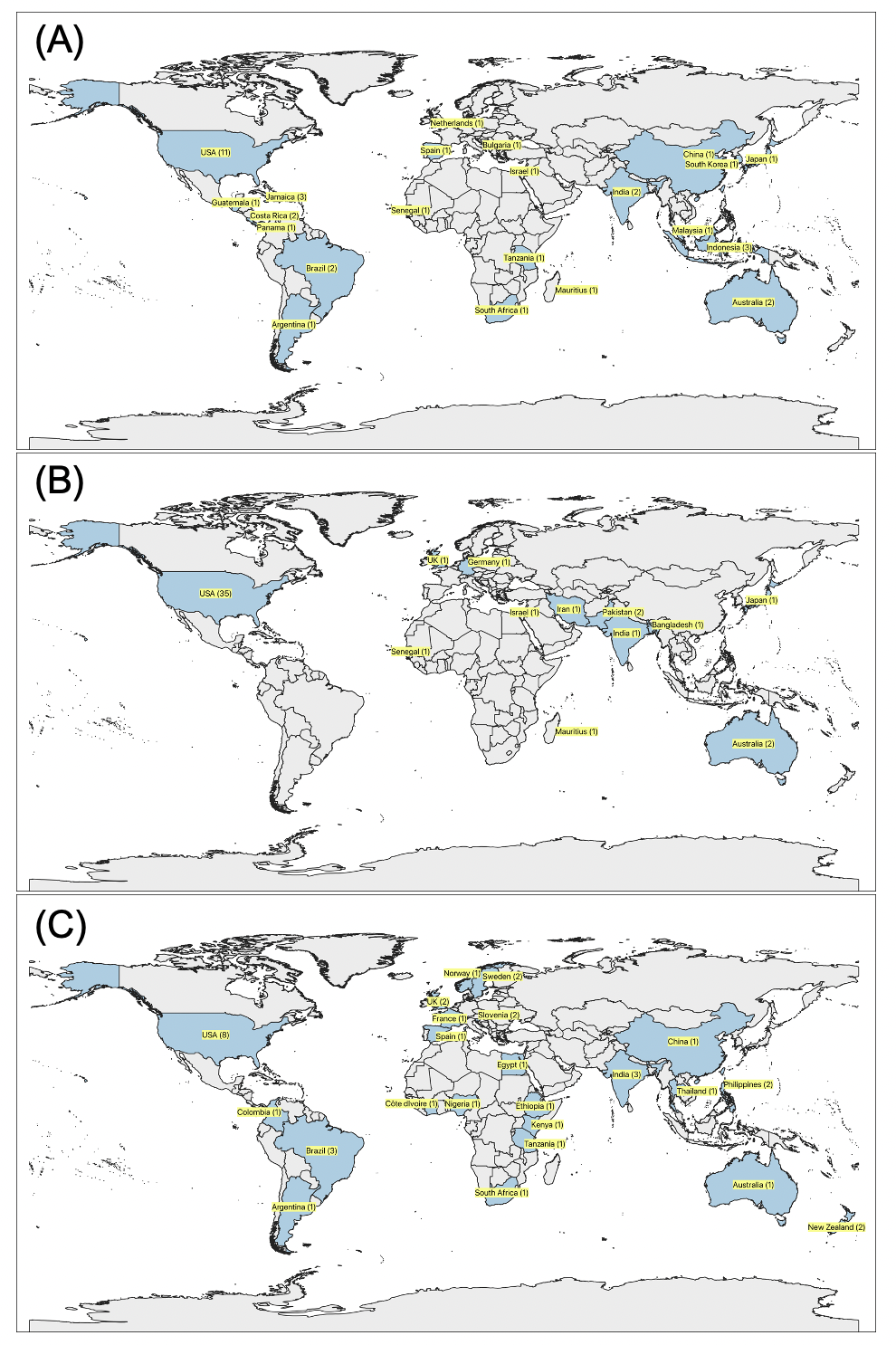
**

**Fig C. Number of studies (in bracket) about (A) exclosure experiments, (B) mitigation experiments, and (C) attitude surveys by countries.** A country with grey background means no relevant study. The base map of country boundaries was from https://www.naturalearthdata.com/.

**
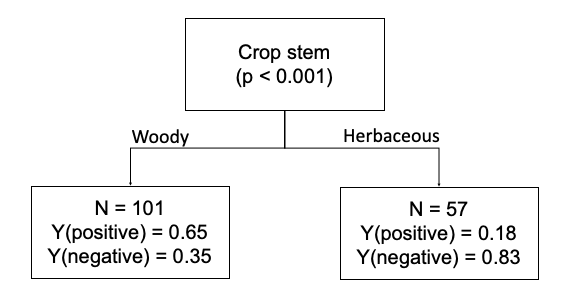
**

**Fig D. Conditional inference tree of the impact of variables on the direction of bird effect (i.e., positive and negative) on crop production**. The conditional inference tree algorithm recursively tests the global null hypothesis of independence between any of the input variables and the response, then select the input variable with the strongest association to the response. The stem type (i.e., woody or herbaceous) is the only significant variable in predicting the direction of the effect size among stem type, food type, climatic region, use of insecticide, use of herbicide, and focal animal.


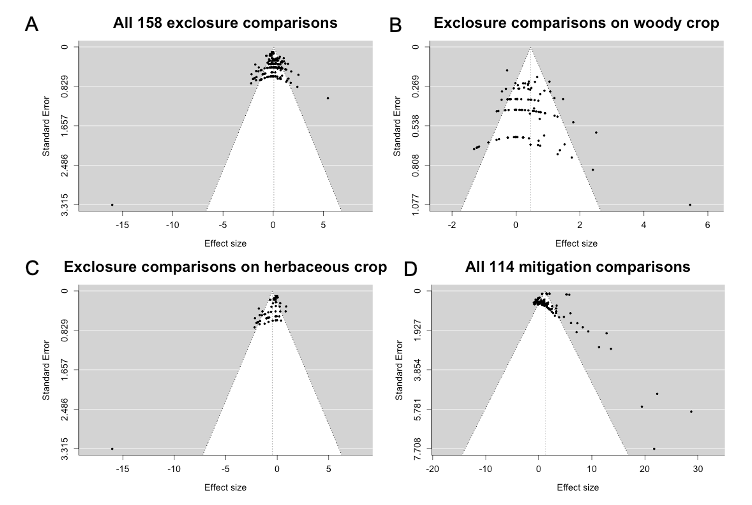


**Fig E. Funnel plots showing the relationship between effect size and standard error of comparisons. A**, for all comparisons of exclosure experiments; **B**, for the comparisons of exclosure experiments on woody crops; **C**, for the comparisons of exclosure experiments on herbaceous crops; and **D,** for all comparisons in mitigation dataset.


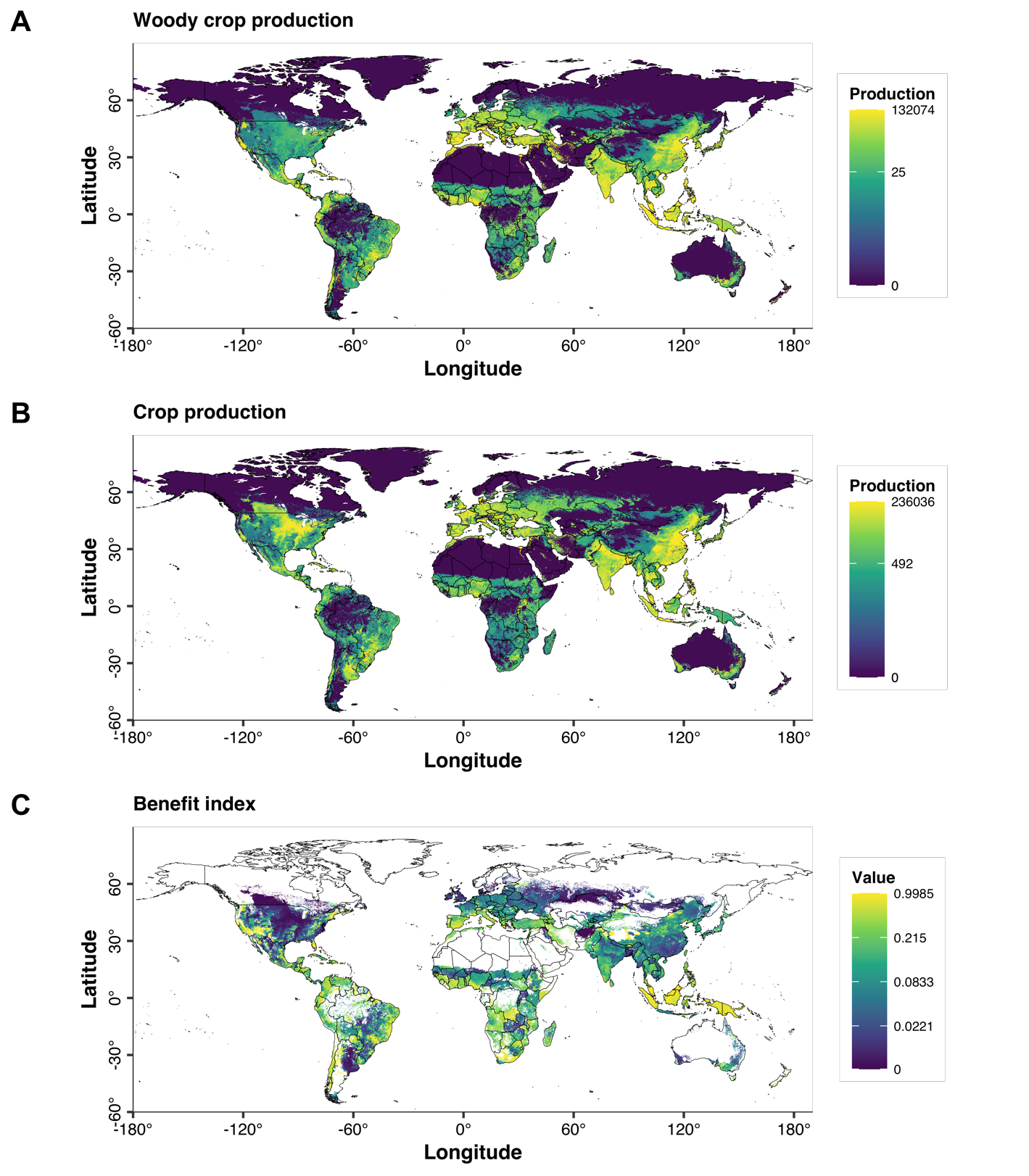


**Fig F. Map of crop production and benefit index. A) Distribution of woody crop production. B) Distribution of total crop production. C) Benefit index,** which was calculated by the proportion of woody crop production **(A)** of total crop production **(B)** in a grid cell. The base map of country boundaries was from https://www.naturalearthdata.com/.


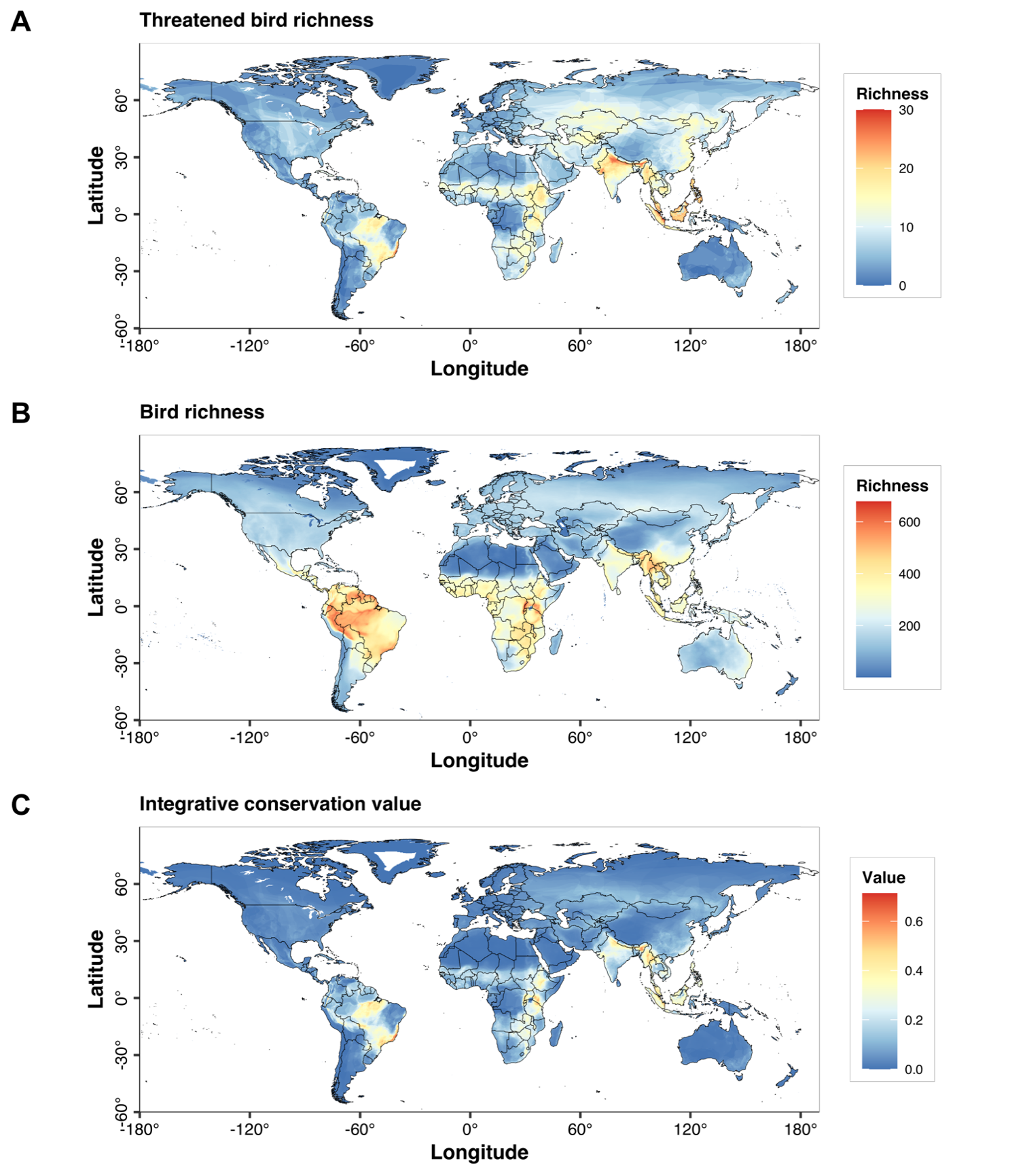


**Fig G. Map of three bird conservation values. The integrative conservation value index (C)** was calculated by multiplying the standardized value of the richness of threatened bird species (**A**), and standardized value of the richness of all bird species (**B**). The base map of country boundaries was from https://www.naturalearthdata.com/.


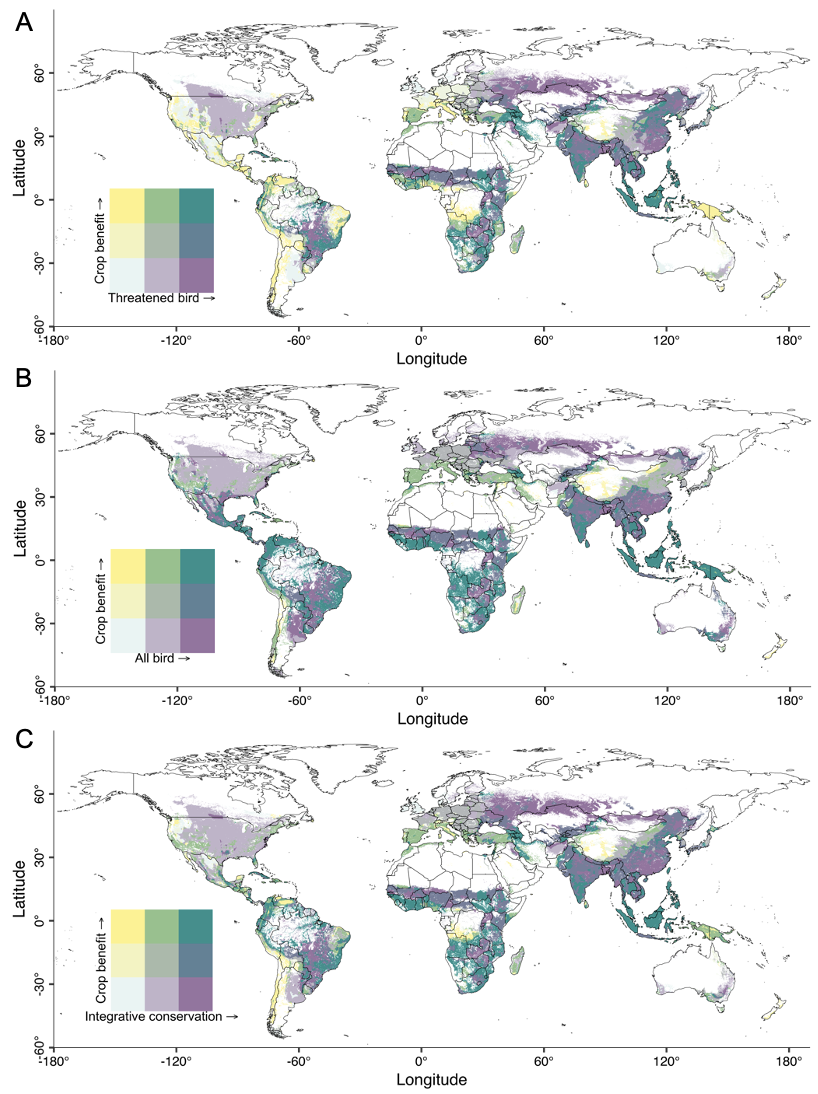


**Fig H. A global mapping exercise of the prioritized areas for efforts to encourage bird-friendly croplands across three conservation value indices.** (**A**), The threatened bird species richness; (**B**) The richness of all bird species; and (**C**) The integrative conservation value index. The bivariate maps are displayed in tercile increments (33%). The dark green color of the bivariate maps (top-right of the legend) represents a higher conservation value and more benefit of woody crops from birds. Areas in white are outside of the prioritized hotspots. The base map of country boundaries was from https://www.naturalearthdata.com/.
