# Supplementary material for: Insights into the coexistence of birds and humans in cropland through meta-analyses of bird exclosure studies, crop loss mitigation experiments, and social surveys
Source: PLoS Biol. 2023 Jul 6;21(7):e3002166. doi: 10.1371/journal.pbio.3002166 (PMC10325107; doi:10.1371/journal.pbio.3002166)
Supplement: Attachment — Submitted filename: response_to_reviewers.docx [file pbio.3002166.s008.docx]

**General reply:**

We are very grateful for the constructive suggestions from the reviewers and editor, Dr. Roli Roberts, that we feel have greatly improved our manuscript. We provide a short summary of our main findings following the reviewers’ input. There are many bird species consuming crops. However, bird can benefit crops by pest control, particularly for woody crops, but also can cause loss to crops in some cases, especially for the herbaceous ones. Therefore, mitigating measures are needed for a better coexistence, and we evaluated their effectiveness. Nevertheless, we found that the services provided by birds are also not fully-appreciated by farmers.

According to the comments and suggestions from reviewers, we have made substantial revision to the manuscript, and the main changes are as follows:

- To clarify the process of the collection of the data from the literature, we have i) added more descriptions in main text and ii) provided all search keywords and rules in the section on ‘Search rules’ in the Supplemental Information.
- To ensure the representativeness of our literature search, we have used the snowball sampling approach. In this revised manuscript, we have combed the non-English literature in the Web of Science. This new search returned 606 papers, but we were able to add only one eligible study in Spanish for the experimental exclosure dataset. Although we acknowledged that it may be important to search for relevant literature in non-English databases like SciELO and J-Stage, our experience is that most of the high-quality and eligible papers of meta-analysis are indexed in Web of Science so we believed that our literature search is relatively representative.
- With regards to the extensive suggestions on our analysis, we have re-analyzed and updated all related figures and tables. However, the suggested changes for our re-analysis did not affect our major findings. Besides, for reproducing our analyses and for transparency, we have provided the raw data, raw codes, and results. Please kindly refer to Supplementary Data 1-4 and the Supplementary HTML file, which illustrated the major analysis flow of this study.
- To clarify the numerous methodological confusions in the manuscript, we have inserted more method descriptions in the main text.
- We have also expanded on our manuscript and provided more detailed discussions.

From our findings, we believe that our work is reliable and robust, and a key strength of this study is its multi-dimensionality. Meanwhile, we acknowledged that there is an important trade-off between the grain and scale of the studies. After accepting many suggestions from the reviewers, the flow among the multi-parts of our work is much better.

**Point-to-point reply (highlighted):**

Dear Ming,
Thank you for your patience while your manuscript "Coexisting with birds in cropland: Insights from multiple meta-analyses" was peer-reviewed at PLOS Biology. Your study has now been assessed and discussed by the PLOS Biology editors, an Academic Editor with relevant expertise and four independent reviewers. Please accept my apologies for the extreme delay incurred due to the failure of our system to flag the prior reviews, and extensions granted to two of the reviewers.

As you will see in their reports at the end of this email, while all four reviewers are impressed by the ambitious nature of your study, they each raise significant concerns about your methodology and the strength of support for some of the claims.

Specifically, reviewer #1 has severe concerns about the methods, and that resolving them may affect the conclusions. Reviewer #2 expresses skepticism around the possibility of publication bias and the purported mechanisms, and is ultimately unconvinced. Reviewer #3 wants clearer framing (including hypotheses), use of non-binary diet data, and worries about biases (especially geographical ones). Reviewer #4 has a number of substantial queries about the stats, the methodological reporting, and has a large number of textual requests.

Reply: Thanks for your summary. We have revised the manuscript accordingly, please find the details in the point-to-point replies below.

Based on these reviews, I regret that we will not be able to invite a straightforward revision of the study, as successfully addressing the concerns raised would entail a significant amount of work (beyond what could be reasonably expected of a revision) and whether the current conclusions would be supported is unclear. 

IMPORTANT: Because producing the required data would require substantial cost and effort with an uncertain outcome, we cannot in good faith invite revision of your study. However, given our and the reviewers' interest in the topic, if further work were to allow you to comprehensively address the reviewer concerns outlined above, we would be willing to consider an extensively revised manuscript as a re-submission. The article would receive a new number and submission date and we would evaluate it in view of any related work published in the interim, but we would make every attempt to engage the same Academic Editor and reviewers in its assessment, if no novelty concerns arise.

Reply: Thanks for the opportunity to revise this manuscript.

IMPORTANT: The Academic Editor asked me to communicate the following points [lightly edited], which may prove helpful in revising your manuscript: "To some extent, methodological limitations are sort of inherent to this type of work so it is important to be as upfront and transparent as possible. I think that is what the reviewers are largely requesting?

Reply: To ensure that our analysis is reproducible and transparent, we have provided the raw data, raw codes, and results. Please refer to our Supplementary Data 1-4 and the Supplementary HTML file, which illustrated the major analysis flow of this study.

Regarding reviewer #2's comments, I do think there is a good point here about causality and conceptual grounding for some of the results that we should communicate back to the authors that they should address in revision. The comment about tree crop eating birds causing tree crop production to increase is agreeably baffling and could just be because like reviewer #2 says, places that support more woody crop production have environmental conditions that support more woody crop eating birds. Presumably that could even be teased apart with some additional analyses?"

Reply: We would like to clarify this concern from reviewer #2. In our work, we actually show that the experimental exclosure of birds (mainly referring to perching birds) to woody crops will decrease crop production. The observed net crop production suggests that the inferred mechanism would be through the benefit of pest control that outweighs the direct consumption by birds. Besides, we also tested the influence of different types of landscape variables (or the environmental conditions) available from the experimental comparisons reported from our numerous case studies. We show that, in a simple counting summary analysis, the effects of the landscape variables (e.g., distance to edge, and distance to or coverage of primary forest or habitat patch) on the crop production, as analysed by the authors, were not significant for two-thirds (66.7%) of the variables. This suggests that environmental conditions were not that influential in affecting crop production. These results are now reported in the main text and Supplementary Table S5.

We appreciate that the scale of the requested additional work is significant and understand that you may prefer to pursue faster publication of this work elsewhere. At this point, your manuscript is no longer under active consideration at PLOS Biology. We might be able to facilitate a faster consideration at another PLOS journal; if this is of interest to you, please let me know.

Please feel free to contact me if you would like to discuss next steps for your manuscript. If you do make the choice to revise and re-submit to PLOS Biology, please provide a point-by-point response to the reviewers' comments when re-submitting. You should include this as a separate file, labelled 'Response to Reviewers'. Please also provide the tracking number of this manuscript [PBIOLOGY-D-22-01542R1] in the 'previous interaction' section of the online submission form and in your cover letter.

I am sorry that we cannot be more positive on this occasion. I thank you for having considered PLOS Biology for publication and hope that you will find the reviewers' feedback helpful as you consider how to proceed with this work.

Sincerely,

Roli

Roland Roberts
Senior Editor
PLOS Biology
rroberts@plos.org

**Reviewer #1:**
This paper works on a very important topic. What the authors are trying to do is very ambitious. Personally, I have never seen any study that covers three different topics on human-bird coexistence in croplands in such a comprehensive manner involving the development and analysis of three global databases.

Reply: Thanks for your positive comments here.

However, I have multiple concerns especially over methodologies. Some of those issues could influence the main conclusions of the paper so I need to see the authors' responses to those concerns before I decide if this study is scientifically robust or not.

Reply: We have revised our manuscript thoroughly, particularly about the methodologies. Please refer to our point-to-point replies.

My concerns are largely fourfold. First, I am concerned about how systematic reviews were conducted, and how the surveys on the public's attitude/perception were identified/collected. There are now established guidelines on how to do systematic reviews, and when compared to those guidelines, this manuscript provides far less information on search strategies, eligibility criteria, and so on (see my comments on L332-341). Therefore in its current form I can't decide if the collected studies represent robust samples.

Reply: To further clarify our process of collecting the literature, we provided i) more descriptions in main text and ii) listed all the search keywords and rules in the ‘Search rules’ of Supplemental Information.

To ensure the representativeness of our literature search, we have used snowball sampling approach in this study. In this revised manuscript, we have combed the non-English literature in the Web of Science. This new search returned 606 papers, but we were able to add only one eligible study in Spanish for the experimental exclosure dataset. Although we acknowledged that it may be important to search for relevant literature in non-English databases like SciELO and J-Stage, our experience suggested that most of the high-quality papers of meta-analysis are indexed in Web of Science so we believed that our literature is relatively representative.

With regards to the suggestions on our analysis, we have re-analyzed and updated all related figures and tables. The suggested changes for our re-analysis did not affect our major findings. Besides, for the ease of our readers to reproduce our analyses and for transparency, we have provided the raw data, raw codes, and results. Please kindly refer to Supplementary Data 1-4 and the Supplementary HTML file, which illustrated the major analysis flow of this study.

To clarify numerous methodological confusions in the manuscript, we have inserted more method descriptions in the main text.

Second, the aim of the spatial prioritisation is unclear. I understand what the map (Fig 6) means but it is unclear what the authors believe should be done in those regions identified, as there is no discussion (see my comments on L265 and Fig 6).

Reply: Based on the findings from our meta-analysis and related scientific evidence from the socio-ecological literature, we uncover the following: i) birds can benefit woody crops but often cause damage to herbaceous crops; ii) many non-lethal measures are effective in reducing the disservice from the birds but do not completely eliminate it; iii) about 40% of the respondents interviewed in our select case studies do not believe that birds could benefit crops; and iv) programs linked to short-term economic benefits appear to have a higher adoption rate of sustainable practices. As such, we propose a win-win strategy for promoting human-bird existence in croplands, the regions broadly identified on the map are the spatial priorities where the woody crop and bird ranges overlapped. This broad spatial mapping prioritization would further require that the relevant conservation and agriculture sectors communicate the evidence of the birds’ benefits to woody crops, to promote the utility of birds when training agricultural producers to reduce crop loss using more bird-friendly (or non-lethal) measures. Therefore, we believe a variety of actions contributing to this strategy should be conducted. To this end, we have added some discussion on this to the main text.

Third, discussions on some important points are missing. These include consequences of geographical bias/gap in identified studies (see my comments on L290-291), the long-term effectiveness of non-lethal mitigation measures considering habituation (see my comments on L294-297), and what causes the difference in the public's perception and attitude between regions and among species.

Reply: Thanks for your comments. To address some of the geographical limitations of our study, we have added a map (Figure S3) to illustrate the uneven patterns of all qualified studies (i.e., exclosure experiments, mitigation experiments, and attitudinal surveys) across the world.

**S3 Fig. Number of studies (in bracket) about (A) exclosure experiments, (B) mitigation experiments, and (C) attitude surveys by countries.** A country with grey background means no relevant study. We did not use the full name of the countries in the map.

As per the reviewer’s suggestion, we have added more to the discussions, especially for the mitigation and attitudinal studies, as follows:

“Third, we collected relevant literatures from three important topics of conservation biology: ecosystem services of wildlife, mitigation of wildlife disservice, and human attitudes toward wildlife. All three topics have at least a publication history over a few decades. Our temporal analysis showed that only studies about mitigation are not increasing (Fig 7 and Supplementary Data 5). This is even though we are in an era of literature explosion [66]. Furthermore, it is widely recognized that variations of effectiveness across sites and habituation are two major challenges for many kinds of technical mitigating measures for vertebrates [14, 67-72]. However, the research efforts on mitigations are particularly spatial-biased with over 70% of the studies (N = 35 out of 48) being conducted in a single country (i.e., the USA) and these experiments were mainly conducted during one crop season. These research patterns may impede the implementation of effective evidence-based solutions and building of social capacity at local scales. On the other hand, we found that the animal behavioral-based measures (e.g., scaring model and bio-sound) appear to be quite promising. As such, more studies on animal senses, including visual, auditory, and odor, are needed to inspire and innovate new measures and to improve upon the traditional ones [73-75].

Fourth, based on 39 independent surveys across the world, we found that the proportion of respondents having a positive attitude toward birds (67%) is higher than that of damage-causing large mammals (46%) [47]. This difference potentially signals that human-bird coexistence may be relatively less-challenging than that of human-large mammals. Meanwhile, the general patterns of attitudes among stakeholders and focal taxa are quite similar [47, 76]. That is, urban residents tend to be more positive towards animals, likely because these animals have a low impact on their livelihoods compared to those in the rural areas; and the attitudes are more negative towards conflict-prone species than less conflict-prone ones, which caused losses to humans. In our study, we found that the perceived disservices from birds are different due to the economic status of involved countries, that may be related to wealth, education, livelihood, and extent and severity of these conflicts [47, 55, 77]. Nevertheless, we did not have enough data to explore the interactions among these social factors. Further, for taking actions at the local scale, studies would need to understand how multifaceted social factors (e.g., experiences, beliefs, and social norms) affect the attitudes and behaviors drawing from existing social and psychological frameworks [27, 78-80].”

Lastly, the collection of data on the public's attitude is not clearly explained (see my comments on L383-387 and L439-441).

Reply: The data collection process of our public’s attitude dataset is similar to those of exclosure and mitigation experiments. To clarify the process of literature collection, we provided i) more descriptions of the process in the main text and ii) listed all search keywords and rules in the ‘Search rules’ of Supplemental Information. The details are provided in the specific comments below.

I have also raised a number of potential issues so please find my specific comments below.

Reply: Because some of the specific comments overlapped with the above-mentioned four key comments, we will refer to them when addressing the specific ones below.

Introduction is very well written, properly citing relevant references.

Reply: Thank you for your positive assessment.

Fig 2b: Please show the resolution of grid cells used.

Reply: This map is the overlay of 1157 polygons of bird distribution range. Here, we set the cell resolution as 10 km to match the resolution of the crop production rasters (i.e., Fig 6). We stated the cell resolution in the ‘Prioritize such efforts in tropical regions’ section.

L134: Avoid arguing "we found that the presence of birds increased crop production", given that the overall mean effect size was not significant. I suggest simply admitting it's not significant overall, but significant in opposite directions for woody and herbaceous crops.

Reply: Thanks for your suggestion. Yes, we have taken the reviewer’s point and revised the statement thoroughly.

Fig 3a: I suggest plotting overall mean effect size and mean effect sizes for woody and herbaceous crops as well as their 95%CI.

Reply: Thanks for the suggestion. We have now plotted these details and added captions for these figures as seen in the following edited figure:

L171-174: "it is possible that habitat alteration …. May have jointly contributed to the observed negative effects" - this was not very clear to me. Could you please elaborate why habitat alteration etc may have explained the negative effects?

Reply: Thanks for pointing this out. We now made some changes to this statement. First, we have now moved this statement to the section of “More research efforts on the coexistence are needed”. Second, we have provided some examples to support our point. Third, delving into the underlying mechanisms would require a proper treatment as it is an extensive and active area of research, so we did not attempt to make detailed discussions here, instead, we cited some relevant studies. The revised discussion is as follows:

“Second, at the local level, our study shows that birds have a generally positive effect on woody crops but also can cause losses to crops in some cases, especially for herbaceous ones in non-tropical regions. These complex patterns may jointly be contributed by habitat alterations (e.g., the common crane *Grus grus* selectively used arable land for foraging around small protected areas) [61], animal traits (e.g., a greater extent of conflict is often related to diet generalists like red-winged blackbirds *Agelaius phoeniceus* and the tolerance for birds, as indicated by flight distance, are higher for species with larger flock size and smaller body size) [55, 62, 63], and human behaviors (e.g., less harmful behaviors towards many species) [64]. These factors also contributed to the human conflicts with large mammals, such as elephants, carnivores, and primates [5, 64, 65]. Thus, a deeper understanding of how multifaceted factors shape the foraging strategies at a species- and community -level is critically needed for tailored managements for birds, especially in agricultural fields with herbaceous crops in biodiversity-rich tropics.”

L199-202: Avoid arguing net and sound are effective as their 95% CIs overlap with zero.

Reply: The averaged effect sizes of net (1.04, -0.17 to 2.25) and sound (0.98, -0.01 to 1.98) are positive but the confidence intervals slightly overlap with zero. For analysis with large sample sizes, we generally do not emphasize the insignificant results. However, the sample size of experiments in field has been relatively small. Thus, and by convention, we generally interpret this result by the averaged effect size and its confidence interval rather than only by its significance test. As such, we stated this result as “are generally effective” in the manuscript.

L234, 238: Are these percentages the mean percentages of respondents with positive attitudes? Or are they calculated from a single pooled dataset? I believe the former would make more sense.

Reply: Thanks for raising this question. The percentage here is the average proportion of respondents with positive attitude across all cases studies. We clarified this in our methods.

Fig. 5. Provide sample size.

Reply: We have provided the sample sizes in parentheses in the following Figure 5.

I also suggest explaining the response variables in each plot in more detail.

Reply: This is a good suggestion. We have added more description in the first paragraph of this section. Please check the details in the main text.

I assume these are the proportions of respondents with "positive attitude", etc but how was "positive attitude" defined using data from different surveys?

Reply: This is another great point. Yes, the specific questions in the surveys are different. Following Kansky et al. work on damage-causing mammals (i.e., Kansky R, Kidd M, Knight AT. Meta‐analysis of attitudes toward damage‐causing mammalian wildlife. *Conservation Biology*), we defined the proportion of respondents who like and favor a species to “increase” or remain “stable” as positive attitudes. Options such as “would like to see more or current-level of geese” and “willing to support efforts to conserve the species” are also considered as positive attitudes. We have added these examples in the main text. Additionally, the specific questions used in surveys were provided in the supplementary file “Supplementary_Data_4_attitude_survey.xlsx”.

L265, Fig. 6: It was not clear to me what kind of prioritisation these areas are for. The green areas essentially mean those areas with higher richness of species (including non-crop feeders) and higher crop benefit from birds. But there is almost no discussion/interpretation about what should be done in those areas.

Reply: Thanks for this point. These prioritizations are for taking actions to improve human-bird coexistence. Based on the findings from our meta-analysis and other scientific evidence from the socio-ecological literature, we uncover the following: i) birds can benefit woody crops but often cause damage to herbaceous crops; ii) many non-lethal measures are effective in reducing the disservice from the birds but do not completely eliminate it; iii) about 40% of those interviewed in our selected case studies do not believe that birds could benefit crops; and iv) programs linked to short-term economic benefits appear to have a higher adoption rate of sustainable practices. As such, we propose a win-win strategy for encouraging human-bird existence in croplands, the regions broadly identified on the map are the spatial priorities where the woody crop and bird ranges overlapped. This broad spatial mapping prioritization would further require that the relevant conservation and agriculture sectors communicate the evidence of the birds’ benefits to woody crops, to promote the utility of birds when training agricultural producers to reduce crop loss using more bird-friendly (or non-lethal) measures. Therefore, we believe a variety of actions contributing to this strategy should be conducted. To this end, we have added some discussion on this to the main text.

What does "efforts to encourage human-bird coexistence" mean? Does it mean conserving birds to increase their ecosystem services for crop production? If so I suspect the authors should use the richness of crop-benefitting species, not all bird species.

Reply: We have rephrased this point as “a better coexistence between humans and birds”.

However, it is not that straightforward when defining what a crop-benefitting species is. Because such data is by and large missing from the individual case studies. To avoid making too many assumptions we hence treated the conservation value of birds (i.e., bird richness) and crop benefit (i.e., woody crop production maps) as two independent measures and use their intersections to highlight areas of potentially high human-bird coexistence priorities.

L267-269: A brief (but thoughtful) discussion on why birds benefit woody crops, rather than herbaceous crops, would be helpful here.

Reply: We regard our partial response to L171-174 as like what is needed for this comment. We would prefer to avoid delving into the underlying mechanisms as it would require a proper treatment as it is an extensive and active area of research, so we did not attempt to make detailed discussions here, instead, we cited some relevant studies as follows:

“Second, at the local level, our study shows that birds have a generally positive effect on woody crops but also can cause losses to crops in some cases, especially for herbaceous ones in non-tropical regions. These complex patterns may jointly be contributed by habitat alterations (e.g., the common crane *Grus grus* selectively used arable land for foraging around small protected areas) [61], animal traits (e.g., a greater extent of conflict is often related to diet generalists like red-winged blackbirds *Agelaius phoeniceus* and the tolerance for birds, as indicated by flight distance, are higher for species with larger flock size and smaller body size) [55, 62, 63], and human behaviors (e.g., less harmful behaviors towards many species) [64]. These factors also contributed to the human conflicts with large mammals, such as elephants, carnivores, and primates [5, 64, 65]. Thus, a deeper understanding of how multifaceted factors shape the foraging strategies at a species- and community -level is critically needed for tailored managements for birds, especially in agricultural fields with herbaceous crops in biodiversity-rich tropics.”

L271, Fig. 6: The definition of "Integrative conservation index" is unclear and needs to be elaborated. For example, how was it calculated, what does a larger value mean?

Reply: Thanks for the comment. We realized that the index was not explained clearly in the main text. Basically, we developed an integrative conservation value index by multiplying the standardized value (value ranges from 0 to 1) of the richness of threatened species and all bird species. We assumed that an area with higher richness of bird species and higher richness of threatened bird species should be of more conservation value. We have now added more description in the main text.

Same for "crop benefit" - how was it measured? Also show values on the x and y axes (eg range and tertile values).

Reply: We regarded a higher proportion of woody crop as more crop benefit from birds, since our results support that birds benefit woody, but not herbaceous, crops. This calculation was based on the global distribution maps of crops obtained from Earthstat (5 minutes by 5 minutes, <http://www.earthstat.org/>). We have also added these descriptions in the main text, as well as shown the values on the axes in the figure.

Fig. 7: I wonder why the authors aggregated the number of studies in each decade's bin. I would suggest simply showing the number of studies in each year as line plots with points.

Reply: In Figure 7, we plotted the number of studies by ten-year bins. This is largely because, as you can see, there are many decade bars that are actually below ten studies, which means that there is less than one study per year. As such, it makes more sense to pool the number in a decadal interval.

Also applying Poisson GLMs should be better than non-parametric tests to see temporal trends.

Reply: Because we used a decade as a unit, we only have 5 data points for each topic. Thus, it is more appropriate to use simple non-parametric tests.

L290-291: This is actually a very important point and I suggest creating maps showing the number of studies in each country for each of the three subfields (ecosystem services, perception, and mitigation) of this study’s focus, and discuss potential consequences of the geographical bias for the conclusion of this study.

Reply: Thanks for your great suggestion. We have now added a map to illustrate the pattern of studies across three subfields across the world. Please see S3 Figure below.

**S3 Fig. Number of studies (in bracket) about (A) exclosure experiments, (B) mitigation experiments, and (C) attitude surveys by countries.** A country with grey background means no relevant study.

As responded above, we have added more discussion about the potential consequences of geographic bias. The text is as follows:

“Third, we collected relevant literatures from three important topics of conservation biology: ecosystem services of wildlife, mitigation of wildlife disservice, and human attitudes toward wildlife. All three topics have at least a publication history over a few decades. Our temporal analysis showed that only studies about mitigation are not increasing (Fig 7 and Supplementary Data 5). This is even though we are in an era of literature explosion [66]. Furthermore, it is widely recognized that variations of effectiveness across sites and habituation are two major challenges for many kinds of technical mitigating measures for vertebrates [14, 67-72]. However, the research efforts on mitigations are particularly spatial-biased with over 70% of the studies (N = 35 out of 48) being conducted in a single country (i.e., the USA) and these experiments were mainly conducted during one crop season. These research patterns may impede the implementation of effective evidence-based solutions and building of social capacity at local scales. On the other hand, we found that the animal behavioral-based measures (e.g., scaring model and bio-sound) appear to be quite promising. As such, more studies on animal senses, including visual, auditory, and odor, are needed to inspire and innovate new measures and to improve upon the traditional ones [73-75].”

L294-297: What I believe is critically missing here is a discussion on the long-term effectiveness of non-lethal mitigation measures. I can imagine that these measures are only effective when first presented, but in the long term the effectiveness will diminish due to habituation. And I suspect most of the studies testing these mitigations only captured the short-term effectiveness. This should be more thoroughly discussed before any conclusion is derived.

Reply: This is a really good point. Yes, while we acknowledged that habituation is a big challenge for many mitigating measures related to large carnivores, marine megafauna, and elephants. We systematically reviewed all studies about mitigation experiments, but most researches were conducted to only test short-term technical measures and, often, only for one crop season. As such, we have added some discussion in the main text and we identified this important issue as a further research direction. Please look under “More research efforts directed to the coexistence” section.

L308: At the moment there is almost no discussion on the public's perception and attitude. It would be important to discuss why attitudes differ among areas of residence, species groups and economic levels, and what the consequence of these differences are for the conservation management of bird species.

Reply: Thanks. We have added more description in the section of “More research efforts directed to the coexistence”. The revision is as follows:

“Fourth, based on 39 independent surveys across the world, we found that the proportion of respondents having a positive attitude toward birds (67%) is higher than that of damage-causing large mammals (46%) [47]. This difference potentially signals that human-bird coexistence may be relatively less-challenging than that of human-large mammals. Meanwhile, the general patterns of attitudes among stakeholders and focal taxa are quite similar [47, 76]. That is, urban residents tend to be more positive towards animals, likely because these animals have a low impact on their livelihoods compared to those in the rural areas; and the attitudes are more negative towards conflict-prone species than less conflict-prone ones, which caused losses to humans. In our study, we found that the perceived disservices from birds are different due to the economic status of involved countries, that may be related to wealth, education, livelihood, and extent and severity of these conflicts [47, 55, 77]. Nevertheless, we did not have enough data to explore the interactions among these social factors. Further, for taking actions at the local scale, studies would need to understand how multifaceted social factors (e.g., experiences, beliefs, and social norms) affect the attitudes and behaviors drawing from existing social and psychological frameworks [27, 78-80].”

L322: How were these 89 crops selected for the analysis?

Reply: Thanks for the question. In the study by Monfreda et al. (Monfreda C, Ramankutty N, Foley JA. Farming the planet: 2. Geographic distribution of crop areas, yields, physiological types, and net primary production in the year 2000. Global biogeochemical cycles), the authors mapped the distribution of 175 common crops (e.g., fruits, cereals, forages, and vegetables) across the world. Based on this 175-crop list, we selected nearly all crops in the categories of fruit, treenut, vegetable, cereal, and oilcrop. However, we excluded several of these crops because i) we cannot distinguish whether it is wild or domestic (e.g., figs), ii) some of the terms are not specific, such as “Nut”; and iii) outside the focus of this study, such as sugar cane. In sum, only 89 crops remained. Moreover, according to the suggestion from Reviewer 4, we have combined “cherry” and “sour cherry”, so the final number of crop types used in our analysis is 88.

L332-341: Information on the search/screening processes is critically missing here. I would suggest first reading through Guidelines and standards for evidence synthesis in environmental management (https://environmentalevidence.org/information-for-authors/table-of-contents-page/) and author instructions on how to report systematic review methods in journal Environmental Evidence (https://environmentalevidencejournal.biomedcentral.com/submission-guidelines/preparing-your-manuscript/systematic-review-protocol) and follow suggestions there.

The authors at least need to provide explanations on:

The choice of search terms: what search terms were used why those terms were selected, why do the authors believe these search terms were sufficient, and so on. Illustrating the search strategy based on PICO elements would be effective too.

Reply: Thanks for pointing this out. We actually used the PICO framework (participants, intervention, comparison, and outcome) to search the literature. In our context, we defined the search rules by combinations of animal, experiment/survey method (e.g., exclosure and questionnaire), target (e.g., crop and farmer), and outcome (e.g., production and attitude), such as the combination of ‘bird+exclosure+coffee+yield’ when searching for exclosure experiments.

For the three sub-topics datasets, we initially focused on birds and bats; for the mitigation dataset, we initially evaluate the effectiveness of measures in reducing the loss of crops, fishery, and poultry caused by birds and bats. While the below search keys drew up a longer and broader list of papers, the final meta-analysis was only focused on birds and crops.

We acknowledged the potential bias of Web of Science, so we also snowball-sampled studies cited by relevant papers at the screening stage to ensure sufficient literature coverage.

**Rules for exclosure experiment dataset**

**Rule1** TS=(bird OR raptor OR "*species name of bird*" OR bat)

**Rule2** TS=(exclud* OR exclosure OR exclusion OR net OR wire OR service OR disservice)

**Rule3** TS=(crop OR agriculture OR corn OR sunflower OR tuber OR seed OR maize OR wheat OR *nut OR cherry OR berry OR rice OR soybean OR sorghum OR fruit OR orchard OR apple OR vegetable)

**Rule4** TS=(yield OR output OR product* OR los* OR biomass OR damage)

**Rule 5 Exclusion** TS=(broiler OR clinical OR muscle OR metabolism OR dairy OR immun* OR cell OR nerve OR genetic OR genomic OR poultry OR egg)

**Rules for mitigation dataset**

**Rule1** TS=(bird OR raptor OR "*species name of bird*" OR bat)

**Rule2** TS=(conflict OR damag* OR feed OR raid OR consum* OR predat* OR kill* OR prey)

**Rule3** TS=(crop OR agriculture OR corn OR sunflower OR tuber OR seed OR maize OR wheat OR *nut OR cherry OR berry OR rice OR soybean OR sorghum OR fruit OR orchard OR apple OR vegetable OR fish* OR pet OR livestock OR chick* OR game* OR peasant OR poult* OR grouse OR goat OR cow OR lamb)

**Rule4** TS=(yield OR output OR product* OR los* OR death OR biomass OR mortality OR stock OR attitude OR perception)

**Rule5** TS=(protect* OR manag* OR *control OR mitigation OR mitgat* OR repel* OR compensat* OR guard* OR trap OR scar* OR bait* OR cull* OR avers* OR fenc* OR poison* OR shoot*)

**Rule6 Exclusion** TS=(broiler OR clinical OR muscle OR metabolism OR dairy OR immun* OR cell OR nerve OR genetic OR genomic OR poultry OR egg)

**Rules for attitude dataset**

**Rule1** TS=(bird OR raptor OR "*species name of bird*" OR bat)

**Rule2** TS=(farm* OR village* OR suburb OR countryside OR community OR coexist* OR “nature reserve” OR “national park” OR livelihood OR hunter OR public OR crop OR agriculture OR fruit OR orchard OR vegetable OR citizen OR tourist OR visitor OR urban OR city)

**Rule3** TS=(attitude OR perception OR awareness OR belief)

Please check the ‘Search rules’ in our Supplemental Information. In addition, we provided S1 Fig for the PRISMA flow of our meta-analyses.

The choice of search systems/databases: it seems that the authors only used WoS, which is known to cause bias in literature identified. The choice of search systems also needs to be justified. Even for WoS, there are a number of databases from which you can choose for searches. Which database(s) was used for the search?

Reply: Thanks for the comment. We acknowledged the potential bias of Web of Science, especially for the research on the mitigation experiments, so we also snowball-sampled studies by looking for new papers cited by the relevant papers at the screening stage to ensure sufficient literature coverage. We used ‘All Databases’ and added information at ‘Data collection and compilation’ section in our main text.

The choice of languages: I assume the authors only searched in English but again it is known that English-only searches could lead to biased conclusions in evidence synthesis. I understand searching in multiple languages could be challenging but I wonder why the authors did not search in simplified Chinese at least? At least this needs to be discussed as an important limitation.

Reply: Thanks for raising this point. To address the reviewer’s point, we re-searched all non-English literature in Web of Science published before December 2020. This new search returns 606 papers. We check all these papers and added only one new study in Spanish for the exclosure dataset. This goes to show that our search is quite thorough.

The largest database in the Chinese language is CNKI (<https://www.cnki.net/>), we initially searched in CNKI but found just one ineligible study of mitigation experiment and no study of exclosure experiments for birds. Therefore, our search relied solely on the Web of Science and snowball-sampling. We have added this information in the ‘Search rules’ section in our Supplemental Information.

Eligibility criteria: those are roughly explained on P21-23, but still need to be more clearly/systematically defined. Again I suggest using the PICO-framework to explain these (eg see Box 3.2 here: <https://environmentalevidence.org/information-for-authors/3-planning-a-cee-evidence-synthesis/>)

Reply: Yes, as mentioned above, we actually used the PICO-framework to define our search rules. Please check under ‘Search rules’ in Supplemental Information.

L344: Why did the authors only include before-after or control-impact designs? What about BACI or randomised controlled trials? These are recognised as more robust designs than before-after and control-impact.

Reply: Thanks for picking this up. This is a misstatement. There are actually many randomized controlled trials in the dataset, we have now rephrased this sentence.

L350, 368: It is unclear to me what kind of measurements in each study were considered eligible as effect size and how effect size from each study was retrieved/calculated. Please elaborate more.

Reply: Thanks for making us clarify this. First, there is no universal measurements for crop production for all case studies; the measurements are usually area-based production, weight of fruit or seed, and within-plant production, such as yield gram per square meter, kilogram per tree, and the number of shoots per square meter.

Second, we would need to retrieve the sample size, mean, and standard deviation for both the control and treatment groups. Since a study may report data for different experimental sites and measurements, multiple data points could be calculated from each study. We then used the equation provided in the main text to calculate an effect size. This has been elaborated in the methods in the main text.

L368: Does this mean the authors only included either before-after or BACI designs? If so why were studies comparing treatment and control (control-impact design) excluded for the mitigation dataset?

Reply: We have responded to this point above.

L380: L223 says the dataset includes 40 independent surveys from 25 countries. How were these surveys identified and collected? No information is provided on those processes. Just like the systematic review of literature, it is critically important to prove that these surveys were systematically identified/collected in as an unbiased and comprehensive way as possible. Otherwise it could be viewed as cherry-picking.

Reply: Thanks for this comment. We used similar methods to search for these surveys as the exclosure experiments and mitigation experiments. In our revised manuscript, we provided all search terms (we have listed them above), please check under the ‘Search rules’ in our Supplemental Information. We believe that our method is quite comprehensive and not cherry-picking.

L383-387: The description of data collection is unclear to me. As far as I understand, these surveys are independent to each other, meaning people's attitude or perception was measured in a different way among surveys. How exactly did the authors define "positive attitudes" from such varying measures? Showing the relevant question of each survey and explaining how positive attitudes were defined from the question would be useful.
Reply: We have provided our response to a similar issue above.

L435-436: The funnel plot for mitigation pairwise (S3 Fig) shows a clear asymmetrical pattern, so I believe this is a classic example of a strong publication bias. But this is not discussed in the manuscript.

Reply: Thanks for pointing this out. Yes, based on the funnel plot, the asymmetrical pattern is a potential bias for the mitigation studies. The good news is that we have used multiple ways to evaluate the publication bias. In our work, if an averaged effect size is significant as indicated by the original data and the funnel plot and/or ranking correlation shows potential publication bias, we would follow-up with the ‘trim and fill’ method as a sensitivity analysis. It recalculated the estimated average effect size by trimming the smaller comparison from the positive side and filling it or mirroring the negative side of funnel plot thereby removing funnel asymmetry issue. Nevertheless, the recalculation did not affect the direction of the effects. For more details, please refer to S3 Table.

L439-441: I found this analysis potentially problematic. This is partly because the data structure is unclear to me.

Reply: Thanks for raising this issue. We have listed the data structure (in partial) in the following form:

| Study | Question type | Question | Proportion | Stakeholder group | Economic status of country |
| --- | --- | --- | --- | --- | --- |
| 1 | Positive attitude | would like to see more or current-level of geese” | 80% | Rural | High |
| 2 | Positive attitude | willing to support efforts to conserve the species | 65% | Rural | Non-high |
| 2 | Service to crop | Do you think birds will benefit crops | 40% | Urban | High |

Please check “Supplementary_Data_4_attitude_survey.xlsx” for more details and the complete data structure.

For example, how many data points were used from each survey?

Reply: Thanks for the question. As you can see in S7 Table, the number of data points from each survey is different. Please refer to S7 Table for details.

If there are multiple samples from each survey, that needs to be accounted for in the analysis.

Reply: Thanks for the suggestion. Yes, while it is better to consider the nested structure of data, the small sample size precluded the use of complex analysis.

Are there any other confounding factors, such as authors, geographical regions/countries, and so on? If the sample size is too small to test the effect of these factors, is it reasonable to derive any conclusions based on such small sample size?

Reply: As mentioned above, we mapped the uneven distribution of studies in S3 Fig. However, we do not consider these listed factors for statistical analysis due to small sample size. Yes, while the questions would be tied to the available data, the small sample size meant that we could not draw any meaningful conclusion about these detailed factors. Instead, we only considered broader categories such as stakeholder type and focal animals, which is a reasonable approach given the tradeoff between the data limitation and the importance of the social dimension of the study.

L445-446: Effect size (the level of benefits) seems smaller (even if not significantly) in the tropics than in non-tropics even for woody crops (Fig 3c). I believe this needs to be accounted for when calculating the crop benefit index; otherwise benefits in the tropics would be overestimated.

Reply: This is a good point. To clarify, we did not compute a crop benefit index that would be sensitive to the differential regional effects. This is because the regional sample sizes are still on the low side so it is critical to avoid under- or over-estimating the crop benefit impacts. As such, we are unwilling to separate the results for broad regions like tropics vs non-tropics. Nevertheless, we acknowledged this potential effect in the discussion.

Another critically important issue here is consequences of geographical bias in studies. Fig 3 shows birds can benefit the production of woody crops, but where are these studies conducted?

Reply: As with above, we mentioned that we added a new map (S3 Fig) to show the uneven distribution of the studies.

If most of the studies were conducted in a certain region, say North-South America, would it be reasonable to apply their findings to other regions, such as Africa? The current manuscripts seriously lacks the discussion of such consequences.

Reply: This is similar to a previous question on geographic bias. We have edited the manuscript based on the reviewer’s comment. First, we added a new map (S3 Fig) to show the uneven distribution of the chosen studies. Second, we revised the manuscript thoroughly to avoid making unreasonable inferences. Third, the lack of studies in certain areas would be regarded as a gap for future researches, and we discussed these gaps under the section on “More research efforts on the coexistence are needed”.

**S3 Fig. Number of studies (in bracket) about (A) exclosure experiments, (B) mitigation experiments, and (C) attitude surveys by countries.** A country with grey background means no relevant study.

L452-453: Why do the richness of all species and threatened species need to be multiplied? Shouldn't it be the richness of those species that actually benefit crop production? The reasoning here is not very convincing to me.
Reply: We assumed that an area with higher richness of bird species and higher richness of threatened bird species should have a larger conservation value. For clarification on this, we have added more description on this in the main text. It is not that straightforward when defining what a crop-benefitting species is. Because such data is by and large missing from the individual case studies. To avoid making too many assumptions we hence treated the conservation value of birds (i.e., bird richness) and crop benefit (i.e., woody crop production maps) as two independent measures and use their broad intersections to highlight strategic areas of potentially high human-bird coexistence priorities.

Reviewer #2:
The authors have undertaken an ambitious meta-analysis of publications relating to bird conservation in agricultural landscapes. I have not seen this attempted before, and I was interested to see the maps of bird species that were vulnerable to persecution based on their dietary preferences. While a previous reviewer mentioned that such maps do not provide on-the-ground, actionable guidance, I think that misses the point of broad-scale mapping work of this kind, which is more strategic than tactical.

Reply: Thanks for your supportive comments. Yes, we think it is important to understand the persecution probability of species with different dietary preferences. However, in the section of “A tenth of all bird species consume crops”, we attempted to provide an overview about the potential human-birds conflicts in croplands for general readers, since birds consume crops directly, that could be deemed as a disservice by local farmers. The reviewer is spot-on again. Our map is more strategic than tactical. For our study, we attempted to give an overview of how to promote human-bird coexistence in croplands and evaluated the effectiveness of mitigating measures to better support on-the-ground actions.

I have many concerns about the breadth and depth of the dataset that the authors have had to rely on in their meta-analyses. To ensure the representativeness of our literature search, we have used snowball sampling approach in this study. In this revised manuscript, we have combed the non-English literature in the Web of Science. This new search returned 606 papers, but we were able to add one eligible study in Spanish for the experimental exclosure dataset. Although we acknowledged that it may be important to search for relevant literature in non-English databases like SciELO and J-Stage, according to our experience of most of the high-quality papers of meta-analysis are indexed in Web of Science so we believed that our literature is relatively representative.

Reply: The other reviewers also have similar issues which we have clarified extensively in our revision.

We used the PICO framework (participants, intervention, comparison, and outcome) to search the literature. In our context, we defined the search rules by combinations of animal, experiment/survey method (e.g., exclosure and questionnaire), target (e.g., crop and farmer), and outcome (e.g., production and attitude), such as the combination of ‘bird+exclosure+coffee+yield’ when searching for exclosure experiments.

For the three sub-topics datasets, we initially focused on birds and bats; for the mitigation dataset, we initially evaluate the effectiveness of measures in reducing the loss of crops, fishery, and poultry caused by birds and bats. While the below search keys drew up a longer and broader list of papers, the final meta-analysis was only focused on birds and crops.

**Rules for exclosure experiment dataset**

**Rule1** TS=(bird OR raptor OR "*species name of bird*" OR bat)

**Rule2** TS=(exclud* OR exclosure OR exclusion OR net OR wire OR service OR disservice)

**Rule3** TS=(crop OR agriculture OR corn OR sunflower OR tuber OR seed OR maize OR wheat OR *nut OR cherry OR berry OR rice OR soybean OR sorghum OR fruit OR orchard OR apple OR vegetable)

**Rule4** TS=(yield OR output OR product* OR los* OR biomass OR damage)

**Rule 5 Exclusion** TS=(broiler OR clinical OR muscle OR metabolism OR dairy OR immun* OR cell OR nerve OR genetic OR genomic OR poultry OR egg)

**Rules for mitigation dataset**

**Rule1** TS=(bird OR raptor OR "*species name of bird*" OR bat)

**Rule2** TS=(conflict OR damag* OR feed OR raid OR consum* OR predat* OR kill* OR prey)

**Rule3** TS=(crop OR agriculture OR corn OR sunflower OR tuber OR seed OR maize OR wheat OR *nut OR cherry OR berry OR rice OR soybean OR sorghum OR fruit OR orchard OR apple OR vegetable OR fish* OR pet OR livestock OR chick* OR game* OR peasant OR poult* OR grouse OR goat OR cow OR lamb)

**Rule4** TS=(yield OR output OR product* OR los* OR death OR biomass OR mortality OR stock OR attitude OR perception)

**Rule5** TS=(protect* OR manag* OR *control OR mitigation OR mitgat* OR repel* OR compensat* OR guard* OR trap OR scar* OR bait* OR cull* OR avers* OR fenc* OR poison* OR shoot*)

**Rule6 Exclusion** TS=(broiler OR clinical OR muscle OR metabolism OR dairy OR immun* OR cell OR nerve OR genetic OR genomic OR poultry OR egg)

**Rules for attitude dataset**

**Rule1** TS=(bird OR raptor OR "*species name of bird*" OR bat)

**Rule2** TS=(farm* OR village* OR suburb OR countryside OR community OR coexist* OR “nature reserve” OR “national park” OR livelihood OR hunter OR public OR crop OR agriculture OR fruit OR orchard OR vegetable OR citizen OR tourist OR visitor OR urban OR city)

**Rule3** TS=(attitude OR perception OR awareness OR belief)

Please refer to more details in the section under ‘Search rules’ in Supplemental Information.

As the authors themselves have noted, the availability of research studies in some areas is concentrated heavily in particular regions, particularly the US. This is not unusual. However, the research seeks to draw global lessons and provide advice at that scale. It is not even certain that the number of studies the authors can draw on from the US are sufficient to permit clear guidance about bird conservation in agricultural regions there.

Reply: As pointed out by this and other reviewers, geographic bias of the studies is an issue. And this bias is most apparent for the mitigation dataset, but not so much for the other two datasets. We have now added a new map (S3 Fig) to show the uneven distribution of the studies and identified this bias as a gap for future researches. The bias in the mitigation dataset is an issue since 73% of studies were conducted in the USA. For the meta-analysis of the mitigating measures, we collected experimental studies and tested the effectiveness of measures by integrating effect sizes across all eligible case studies. Nevertheless, the results were quite limited due to sample sizes and geographical coverage. As such, we were careful not to extrapolate our limited findings to other regions with no case studies. In addition, it is also possible that a measure has inconsistent outcome even among quite similar sites, so we added the notable limitations of our datasets in the discussions on bias. We have edited the manuscript based on the reviewer’s comment. Besides adding Figure S3 to show the uneven distribution of the chosen studies, we have revised the manuscript thoroughly to avoid making unreasonable inferences. The lack of studies in certain areas in the world would be regarded as gaps for future mitigating researches, and we discussed these gaps and dataset limitations broadly under the section on “More research efforts on the coexistence are needed”.

**S3 Fig. Number of studies (in bracket) about (A) exclosure experiments, (B) mitigation experiments, and (C) attitude surveys by countries.** A country with grey background means no relevant study.

Overall, the meta-analyses do not appear sufficiently convincing individually or taken as an ensemble to enable strong conclusions. For example, the authors point out that birds increase the production of woody crops, which seems to mean that fruit production for those crops is higher because there are more birds to consume those crops in those areas. Examination of this suggestion leads to some challenging questions that are not addressed in the manuscript in any transparent way.

Reply: Thanks for raising this to our attention. We respectfully disagree with the reviewer on this point. We presented the largest and most comprehensive exclosure experimental evaluation such that we were able to draw a meaningful conclusion on the effects of birds on crop production across the world. Nevertheless, we realized that there may be some parts of the manuscript that we should improve and clarify. As such, we provided the highlights and changes here. In the “Introduction”, we have described, in detail, how birds could potentially affect the production as follows:

“Birds, distributing across the globe, share lands with humans at a substantial scale and provide diverse provisions (e.g., meat and eggs) and cultural services (e.g., birdwatching and ecotourism) to humans [7, 9]. Also, birds affect crops through direct (e.g., crop consumption) and indirect (e.g., pest control) processes [8, 10, 17]. Thus, birds were often treated as a key indicator taxon of agricultural sustainability in global biodiversity conservation framework (e.g., Convention on Biological Diversity). Most birds prey on invertebrates, including not only herbivore arthropods (e.g., moth larvae and stink bugs) that consume plants but also the predaceous arthropods (e.g., spiders and ants) that predate these pest herbivores [18, 19]. While birds have shown to suppress the herbivore arthropods in wild plants and several crops [17, 20]. Furthermore, birds not only provide pollination services for many plants [21], they also directly caused losses to farmers by consuming crops including cereals and fruits [22-25]. To our knowledge, there is presently a limited number of studies synthesizing evidence to show that the indirect services actually cascade down to crop production [20, 26].”

In our manuscript, counter to the reviewer’s claim, we did not assume “fruit production for those crops is higher because there are more birds to consume those crops in those areas”. In the eligible exclosure experiments, the access of birds to crops was intentionally manipulated (i.e., a binary variable 1 or 0) using grids of proper mesh size, where crops with grids only allow the access of arthropods (see figure below from the main text). Through these well-controlled experiments, our meta-analysis conclusively showed that across all eligible studies the exclosure of the birds (mainly referring to perching birds) to woody crops, but not herbaceous crops, would decrease crop production. This suggests that the most likely mechanism for this net benefit could be due to the benefit of pest control outweighing the direct consumption by birds.

First, there is a lack of transparency about the nature of the experiments in the main text that is key to interpreting the results: are the studies all working with population sizes, diversity (a combination of richness and evenness), or richness? Bird species numbers are not the same as bird population sizes, and the pathway to an impact on fruit set has to go through populations to a greater extent than species. So, there is a logical gap, or at least some uncertainty, there, and going digging through the supplementary materials or primary studies shouldn't be necessary to sort it out. Yet, the challenge is that this finding doesn't appear strongly related to a hypothesis. How would having more tree crop eating birds cause tree crop production to increase? The only pathway I can think, short of invoking a really exotic argument about pollination, is that birds eating more tree crop production would have to reduce the production of the crop. Perhaps there is a contrasting pathway, but I did not see one presented. A more likely interpretation, it seems to me, is that places that support more woody crop production have environmental conditions that support more woody crop eating birds. The generalized tests the authors conducted that could have confounded their analyses seem to nod in the direction that their result is hard to explain, but the tests do not seem powerful to me.

Reply: Thank you for raising these important points. The conduct and context of the experiments are key to understanding the issues we faced. In the experiments, the access of birds to crops was intentionally manipulated using grids of proper mesh size, which allows the access of arthropods. To clarify the experiments, we have now added more descriptions and references in the “Birds often benefit woody, but not herbaceous, crops” section. Many of these experiments simply treated the bird community (mainly referring to perching birds) as an assemblage rather than the use of population size or some diversity index (richness or abundance). While some experimental studies explored how on-farm bird richness and abundance affect the impact of birds on crop production, such as a study on “Natural habitat does not mediate vertebrate seed predation as an ecosystem disservice to agriculture”, they belong to the minority. Most of the experimental studies only reported and quantified the overall outcome of the birds’ effect on crop production, with little known about the exact mechanisms, including the environmental conditions, behind them. Therefore, although the reviewer pointed out interesting questions, we did not have enough data to explore them largely due to the sample studies’ limitations.

Nevertheless, we have stated the possible mechanism issues as limitations in the main text. Some of the possible mechanisms are mentioned in the introduction below. And we avoid delving into the underlying mechanisms in the discussion due to 1) the lack of direct evidence from our studies, and 2) it would require a proper treatment as it is an extensive and active area of research, instead, we cited some relevant studies as follows:

“Birds, distributing across the globe, share lands with humans at a substantial scale and provide diverse provisions (e.g., meat and eggs) and cultural services (e.g., birdwatching and ecotourism) to humans [7, 9]. Also, birds affect crops through direct (e.g., crop consumption) and indirect (e.g., pest control) processes [8, 10, 17]. Thus, birds were often treated as a key indicator taxon of agricultural sustainability in global biodiversity conservation framework (e.g., Convention on Biological Diversity). Most birds prey on invertebrates, including not only herbivore arthropods (e.g., moth larvae and stink bugs) that consume plants but also the predaceous arthropods (e.g., spiders and ants) that predate these pest herbivores [18, 19]. While birds have shown to suppress the herbivore arthropods in wild plants and several crops [17, 20]. Furthermore, birds not only provide pollination services for many plants [21], they also directly caused losses to farmers by consuming crops including cereals and fruits [22-25]. To our knowledge, there is presently a limited number of studies synthesizing evidence to show that the indirect services actually cascade down to crop production [20, 26].”

“Second, at the local level, our study shows that birds have a generally positive effect on woody crops but also can cause losses to crops in some cases, especially for herbaceous ones in non-tropical regions. These complex patterns may jointly be contributed by habitat alterations (e.g., the common crane *Grus grus* selectively used arable land for foraging around small protected areas) [61], animal traits (e.g., a greater extent of conflict is often related to diet generalists like red-winged blackbirds *Agelaius phoeniceus* and the tolerance for birds, as indicated by flight distance, are higher for species with larger flock size and smaller body size) [55, 62, 63], and human behaviors (e.g., less harmful behaviors towards many species) [64]. These factors also contributed to the human conflicts with large mammals, such as elephants, carnivores, and primates [5, 64, 65]. Thus, a deeper understanding of how multifaceted factors shape the foraging strategies at a species- and community -level is critically needed for tailored managements for birds, especially in agricultural fields with herbaceous crops in biodiversity-rich tropics.”

The authors suggest their findings are robust to publication bias, at least for woody crops. I think one of the key publication biases to be concerned about is the "file drawer problem". In other words, studies that do not find expected relationships are published less frequently than those that find a positive result.

Reply: This is a point we need to clarify better here. For the representativeness of literature, we have used snowball sampling approach in this study. For our revision, as explained to the other reviewers, we re-searched non-English literature in the Web of Science. This search returns 606 papers, we check all these papers and added only one eligible study in Spanish in the exclosure dataset. This suggests that we are quite thorough with our search of the literature. We have now also included our detailed search words and terms in our supplementary information.

As with all high-quality meta-analysis, testing the publication bias is a core approach. In our study, we comprehensively assessed the potential publication bias graphically (i.e., funnel plots), numerically (i.e., Rosenthal’s fail-safe number), and statistically (i.e., Kendall’s rank correlation). If the fail-safe number is sufficiently high (i.e., > 5n + 10, where n is the number of pairwise comparisons), the significant results can be considered robust. The rank correlation examines the relationship between effect size and sample size across comparisons. Significant p values indicate publication bias, whereby comparisons with small sample sizes are only published if they show large effect sizes. If the averaged effect size is significant and the funnel plot and/or rank correlation show(s) potential publication bias, we would use the ‘trim and fill’ method as a sensitivity analysis, which recalculated the estimated average effect size by trimming the smaller comparison from the positive side and filling it or mirroring the negative side of funnel plot thereby removing funnel asymmetry. This technique provides an estimate of how the overall effect size would change if we were able to incorporate all missing studies. The ‘trim and fill’ method was particularly required for the exclosure experimental dataset on herbaceous crops, please check the Supplementary Table S3. Please refer to the details about testing publication bias in “Materials and Methods” section.

For potential bird-related damage to herbaceous crops, the authors offer a series of potential justifications for their findings. Parenthetically, it is worth noting that the authors are basically reporting here that birds that are known to eat these crops actually do: studies conducted in places where birds are herbaceous crop pests confirms the expectation. It is useful to report the consensus among those studies and the variation in effect sizes among crop types. The results here are based mostly on temperate-zone research.

Reply: Thanks for the suggestion. In our revision, we have added a new map (S3 Fig) to show the uneven distribution of the studies used in our meta-analyses. For example, we noticed that the negative effect of birds on herbaceous crops in the tropics is not significant, as such we suggested that more research about the outcome of bird community on herbaceous crops in tropical areas is required. Please refer to “More research efforts on the coexistence are needed” section for more details. Besides, we estimated the variable of effect size among crops, please check Supplementary Table S4.

The authors offer a series of speculations regarding the mechanisms leading to their results (around lines 171 and down). I was not able to evaluate any of these mechanisms and I was not sure why they needed to be discussed. The hypothesis around why birds that eat particular things seem to affect the availability of those things seems to be its own mechanism. I am not sure how "fear landscapes" or "animal traits" add to insight and these were not explained.

Reply: The reviewer highlighted a good point. We agreed that this statement may be not suitable for discussion in the original section. In our revision, we have moved this statement to the section of “More research efforts on the coexistence are needed”. In addition, we have provided more contextual appropriate examples. Lastly, we recognized that we need to understand the mechanisms but it is an extensive area of research, so it is outside our scope here to make detailed discussions. Instead, we cited some relevant studies in our revision as follows:

“Second, at a local-level, our study shows that birds have a generally positive effect on woody crops but also lead to losses to crops in some cases, particularly for herbaceous ones in non-tropical regions. These complex patterns may jointly be contributed by habitat alterations (e.g., the common crane *Grus grus* selectively used arable land for foraging around small protected areas) [61], animal traits (e.g., a greater extent of conflict is often related to dietary generalists like red-winged blackbirds *Agelaius phoeniceus* and the tolerance of birds, as indicated by flight distance, are higher for species with larger flock size and smaller body size) [55, 62, 63], and human behaviors (e.g., less harmful behaviors towards many species) [64]. These factors also contributed to the human conflicts with large mammals, such as elephants, carnivores, and primates [5, 64, 65]. Thus, a deeper understanding of how multifaceted factors shape the variations of foraging strategies at a species- and community-level is critically needed for tailored managements for birds, especially in agricultural fields with herbaceous crops in biodiversity-rich tropics.”

The survey summaries about people's attitudes to birds didn't reveal as much as I would have liked, and the survey meta-analysis seemed independent from the other meta-analyses in terms of results. This general view is echoed by other reviews previously. The survey respondents (about 25k people, which is a goodly number) are divided between rural and urban areas. The authors give the sample size in units of numbers of studies, but the weight of respondents might still tilt toward urban, rather than rural. Moreover, it is not clear that the surveys were conducted in areas where birds are agricultural pests, leading to the possibility that people who are not negatively affected by bird effects on crops might swamp the response rates. If so, it is not surprising that most people like birds and the reporting in the paper is not sufficiently clear to enable me to interpret the results thoughtfully.

Reply: Thanks for the reviewer’s comment. The fact is that all three datasets are truly independent. And in reality, very few studies will address all three dimensions concurrently to adequately address the human-bird coexistence issue in the agricultural landscape (see conceptual figure below). Our main rationale for including the attitudinal dataset is so that we can raise cross-sector awareness about the underappreciated human dimension when it comes to developing practical solutions to resolve human-bird conflicts in complex socio-ecological cropland systems.

To address the reviewer’s issue with the dataset, we have clarified more details. In the survey dataset, most of these surveys (71.8%, N = 28) were targeted at villagers or farmers. We also acknowledged that the attitudes may be different because the birds may have little to no impact on some people. Therefore, we defined the stakeholder groups as urban citizens, rural residents (mainly referring to farmers and villagers), and the mix of the two. Nevertheless, we found that the urban citizens (59%) tend to be positive toward birds than that of farmers or villagers (34%). Because specific surveys targeting birds as agricultural pests are lacking, we only attempted to understand the general attitude towards birds and the surveys are not necessarily conducted in areas where birds are pests though we could assume that rural folks and farmers to be more affected by the human-bird conflicts on croplands than those in the urban areas. We have clarified these points and have also stated how we classified people’s positive and negative attitude in the revision.

A previous reviewer noted that this paper should be divided into two or three pieces, enabling a thoughtful consideration of each of its subsidiary meta-analyses. I concur with this view. As it is, I do not feel that the meta-analyses hang together well enough to create a coherent perspective on the work.

Reply: Thank you for your feedback. Yes, our team debated extensively on how best to present our work. We acknowledged that these three datasets are quite independent from each other, however, there just isn’t a unified dataset to answer all these fundamental questions about the human-bird coexistence system. In reality, there existed very few studies that will address all three key dimensions simultaneously to adequately address the human-bird coexistence issue in the agricultural landscapes. In the end, our main rationale for weaving all three datasets together is so that we can more effectively raise cross-sector awareness, particularly about the underappreciated human dimension, when it comes to developing practical solutions to resolve human-bird conflicts in complex socio-ecological cropland systems. There is always a trade-off between the insights obtained due to the grain and scale of a study. Thankfully, two other reviewers are really supportive of retaining both the social and ecological dimensions in our work. Our hope is that by taking in many constructive suggestions by the reviewers, our revision will present a more coherent perspective.

One reviewer noted that the paper needs to have dozens of authors as if this was a requisite for a meta-analysis or for "big picture" research. This suggestion is prescriptive and is outrageously editorial in nature. I disagree with it on several levels and do not believe the authors need to respond to it.

Reply: Thank you for voicing your strong support for us here – we really appreciate it.

Reviewer #3:
Review for Plos Biology PBIOLOGY-D-22-01542R1
Review for coexisting with birds in cropland: Insights from multiple meta-analyses

Dear authors, 

Thank you for giving me the opportunity to review your article: "coexisting with birds in cropland: Insights from multiple meta-analyses". I read your paper with great interest. I have also read the previous review, and therefore my comments are mostly complementary. I believe your work deserves moderate revisions, because I suggest a further revision on the introduction, and a better explanation of the biases and limitations on your analysis. 

Please take my comments in the constructive spirit in which they were intended.

I believe the revised version has taken into account an interesting angle, and I agree with the co-authors that keeping both the ecological and the social dimensions together is important, but not without explaining the biases in the sample.

Reply: Thank you for your support in retaining both the ecological and social dimensions in our study. Based on the other reviewers’ and your suggestions, we have now added extensive explanations about our limitations, bias and the uneven distribution of our datasets, please refer to our point-by-point response below.

Introduction:
I think this introduction needs to better explain and summarize the value of birds to people, and the framing of services and disservices. It never defines what ecosystem services are, what disservices are, or how birds can provide them.

Reply: Thank you for your input. We have now added a brief definition about the service and disservices and more descriptions about the value of birds to people. Please refer to the “Introduction” section.

I suggest reframing the introduction to better include and cite colleagues who publish reviews on avian ecosystem services/disservices. Please take a look at the work by Chris Whelan, and by Chan Sekerciouglu, and please consider citing their work.

Reply: Thanks for your suggesting relevant papers to cite for our study. In fact, we have already cited a paper by Drs Chris Whelan and Cagan Sekerciouglu (“Bird and bat predation services in tropical forests and agroforestry landscapes”). Nevertheless, we have added three more papers: “Ecosystem services provided by Neotropical birds”; “Why birds matter: from economic ornithology to ecosystem services”; and “Ecosystem services provided by birds”.

Lines 74-84: While I appreciate that authors wrote down the research questions, I would like to see the hypotheses they are testing with the predictions in the paper itself. I find it difficult to understand why they designed the analyses the way they did without the hypotheses and predictions clearly stated.

Reply: Thanks for your suggestion. In our setup, we elected to ask four major research questions, instead of stating the hypotheses. This is largely because the nature of a meta-analysis is to evaluate and assess consistent evidence and support across numerous studies. Therefore, it is unconventional and meaningless to simply make a prediction about, say, which mitigating measures are effective or more effective than the others before each analysis as the point is to find out the most effective one(s). Therefore, we preferred to just state research questions.

Methods:
I believe some of the diet in the handbook of birds of the world is not up to date, and I recommend re-doing the analyses with the EltonTraits database https://esajournals.onlinelibrary.wiley.com/doi/10.1890/13-1917.1, which does not treat diet as binary variables (0s and 1s), and instead it treats it as a percentage of diet (e.g., what % of diet of a bird is invertebrate), this would provide a better inference on whether these birds cause disservices, and how much.

Reply: Thanks for your suggestion. We are familiar with the EltonTraits database. While the EltonTraits database breaks the diet composition into percentages, including the percentage of invertebrates, mammals and birds, fish, and fruit etc, their diet data is too coarse for our study. Here, we need to know the crops consumed by the birds. Meanwhile, for each species, the Bird of the World (BoW) provides a “Diet and Foraging” section, that includes the specific crops consumed by each bird species. Please refer to a sample illustration below. As such, we opted to use the BoW database.

Results on "Birds often benefit woody, but not herbaceous, crops"
I think this study has too many biases to make global claims, not only on the types of studies it reviewed, but on how the data were analyzed. If it had reviewed studies in other parts of the world, it would have reported that other families are major crop consumers (finches, woodpeckers damaging tree crops). Moreover, by only looking at studies published in English and those outside the tropics, it is inferring things that are counterintuitive to more local studies. For example, I am suspicious of your finding that states that "globally there are more bird species reported to consume crops in Europe, North America, East Africa, and Southeast Asia than other regions of the world". This is probably more an artifact of how you sampled, and the studies you chose to include.

Reply: Thanks for raising these points. For the representativeness of literature, we have used snowball sampling approach in this study. For the revision, we re-searched non-English literature in the Web of Science. This search returns 606 papers, we check all these papers and added only one eligible study in Spanish in the exclosure dataset. According to our extensive experience of reading many papers for the meta-analysis approach, the representativeness of this study is the best possible.

Please note that this particular meta-analysis is based only on the exclosure experiments. In these exclosure experiments, the access of birds to crops was intentionally manipulated (a binary variable 1/0) using grids of proper mesh size, which allows the access of arthropods but not the birds. Therefore, we could evaluate the net effect of birds on crops. On the other hand, we acknowledged that we uncovered many papers that reported the damage by birds but did not evaluate their effect in an experimental way. As such, our global evaluation is limited by the eligible studies and we have now emphasized this caveat in the text. We carefully interpreted our results from Figs 3 and 4 as “In sum, birds have a generally positive net effect on woody crops but negative cases also exist.

Furthermore, we added a new map (S3 Fig) to illustrate the uneven pattern of studies across countries. In our dataset, the number of comparisons for woody crops in tropics and non-tropics is quite similar which makes our ‘woody crops’ finding robust, while for herbaceous crops, most data are from non-tropics. Nevertheless, because the negative effect of birds on herbaceous crops in the tropics is not significant (Fig 4c), we propose to carry out future research on the outcome of bird community on herbaceous crops in tropical areas. Please refer to the section on “More research efforts on the coexistence are needed”.

To understand the spatial extent of the potential direct (seen) disservices, we identify crop-consuming species by evaluating all the diet-related descriptions in the Bird of the World (BoW), which is a comprehensive and widely-used database about bird life history at a global scale. For each species, the BoW provided a specific “Diet and Foraging” section, including the specific crops consumed by birds. As such, with the BoW as an authoritative source, we stand by our statement on “In general, the crop-consuming birds are widely distributed, with higher richness reported in Europe, North America, East Africa, India, and Southeast Asia than other regions (Fig 2b)”. Nevertheless, despite the BoW being the most comprehensive and widely cited document on the life histories of birds, the diet descriptions may need a revision, since it is possible that the lack of field observations for some species and the reporting preference of some authors may generate some bias. Therefore, we suggest that future updating efforts could pool other reputable databases.

If for example, you included studies in Spanish, you would realize that in South America and Central America lots of species consume crops, especially since South American countries hold the highest species richness in the world, and their economies are also based in agriculture, and particularly small-holders' led agriculture.

Reply: This is a great point. Our results are highly dependent on our datasets and, as mentioned above, there is a lack of Spanish language papers that made our criteria for the meta-analysis. For the broad purpose of generating strategic prioritizing maps, we identify crop-consuming species by evaluating all the diet-related descriptions for all recorded species in the Bird of the World. Because of the high richness of crop-consuming species in South America, it is not surprising that we also find this region to be highly prioritized. In fact, high richness is strongly correlated with high crop-consuming species richness across the globe. Nevertheless, we cannot seem to find enough eligible Spanish papers to evaluate this relationship in the South and Central American region. Therefore, we discussed this potential limitation under the section of “More research efforts on the coexistence are needed”.

I think, you need to explain the caveats of your analysis, the regional bias in the studies that you are choosing to review, and the fact that these studies on crop damages are more likely to be reported in native languages, which are skewing your results. The same bias applies to the results of the section "more research efforts in conservation are needed", where you also show that 70% of the studies are from USA.

Reply: Thanks for your suggestions. These issues have been raised by the other reviewers and we have added more discussion on how we combed the literature (including non-English ones), and about the uneven distribution of studies among our three global datasets.

Methods:
Line 353- I don't understand the logic of using bats, please explain- or drop these studies because they do not seem to fit with the rest of the analyses, the title of the paper, or the purpose of this paper.

Reply: Thank you for bringing this up. In all exclosure experiments, we tried to only choose papers where birds are the focal animals of interest, as defined by the original case studies. Yet, some experiments were not clear as to whether leaf-gleaning bats were excluded. For these papers, we cannot separate the effect of birds from bats. Instead, to account for this effect statistically and to retain as many eligible papers as possible, we used ‘Focal animal’ as a fixed variable in the model to evaluate if our results were confounded by the bats. Fortunately, we did not find the overall effect to be affected by this ‘Focal animal’ variable. Please see Fig 3d and the caption. As such, it is necessary to consider bats for our comparisons.

One of the key assumptions for the analyses presented in Fig 5 is that the stakeholder groups are independent from one another. I do not believe this is the case, where you have a group that is urban, another that is rural, and another that is urban/rural. I believe this is inappropriate because the urban/rural one could be anything, also urban could have so many subdivisions, and rural as well. I do not see how these results are informative or help better understand the contexts for human-wildlife conflicts or coexistence.
Reply: Thanks for your comment. This is a similar point raised by another reviewer. We have clarified the structure of our attitudinal survey dataset in our revision. These social surveys were often conducted in different countries (please see the S3c figure below) and so the data is independent. In addition, we have rephrased the statement to “We classified the focal stakeholders as urban residents, rural residents (mainly referring to villagers and farmers), and the mix of the two.” We agreed that this may not be a perfect classification as this is based on the assumption that most of the farmers are living in rural areas, for example. But this is one that is most feasible given the diverse nature and limitations of the data. By using the three ‘broad’ groupings, we hope to gain some broad insights and to draw some generalizations from our findings. For instance, we found that although pest control service offered by birds is widely recognized in global biodiversity conservation agreements, only 61% of the rural residents thought that birds could benefit crops. Thus, it appears that on the ground lots of rural residents treat birds as pests. These insights are particularly useful when developing practical solutions for promoting human-bird crop existence in croplands.

**S3 Fig. Number of studies (in bracket) about (A) exclosure experiments, (B) mitigation experiments, and (C) attitude surveys by countries.** A country with grey background means no relevant study.

Minor
Line 62: should be "observe" instead of "observed"

Reply: We have revised this.

Line 78: question iii should be split in two, one about where the disservices exist, and another about which nonlethal mitigation measures are effective

Reply: We have taken up your suggestion and revised this.

Line 161-162: The toxicity of pesticides is still reported to be harmful to birds nowadays, see de Montaigu, C. T., & Goulson, D. (2020). Identifying agricultural pesticides that may pose a risk for birds. PeerJ, 8, e9526.

Reply: We have deleted the related statement.

Line 175: "Experiments" typo

Reply: We have corrected the typo error.

I agree that using the Tobias dataset is not needed; I also agree that not including many coauthors is ok.

Reply: Thanks for your support.

I echo the concerns of the associate editor about making global claims based on this mapping exercise, for the reasons I state above.

Reply: We have made extensive changes to the manuscript thoroughly and we hope that by addressing all the constructive comments from all the reviewers, our revised and improved manuscript is much improved.

Reviewer #4:
Huang et al. conducted a large meta-analysis on social and ecological dimensions of birds in agricultural systems. They examine net impacts of birds on crops, efficacy of management options, producer attitudes towards birds, and then identify priority areas for co-managing birds for ecosystem services and conservation. 

I want to congratulate the authors on undertaking such a large and ambitious project! The work undertaken was quite a large effort. To my knowledge, prior meta-analyses have been done on the production end, but it is novel to synthesize all the socioecological components this study did. I think the manuscript has a lot of very interesting/useful information that will be relevant to a lot of ongoing research across avian agroecological systems. I do, however, have some concerns and suggestions.

Reply: Thank you for your very positive comments.

It is extremely challenging to conduct and synthesize these big, interdisciplinary meta-analyses. The first challenge is acquiring and sorting through papers. Due to sparse details on the search protocol, it is hard to assess this phase.

Reply: The other reviewers also brought up this point. As such, we have clarified the process of our data collection. We have added more descriptions of our process in main text and also provided all search keywords and rules under the section in the ‘Search rules’ in the Supplemental Information. For the representativeness of literature, we have used snowball sampling approach in this study. For this revision, we re-searched non-English literature in the Web of Science. This search returns 606 papers, we check all these papers and added only one eligible study in Spanish in the exclosure dataset. We have since re-analyzed and updated all related figures and tables in our revision. The re-analysis did not affect our major findings of this study. Besides, for the ease of reproducing our analysis and to promote transparency, we have provided all the raw data, raw codes, and results. Please consult Supplementary Data 1-4 and the Supplementary HTML file, which illustrates the major analysis workflow of our study. To clarify many of the methodological confusions in the manuscript, we have moved more method descriptions to more prominent sections of the revised manuscript.

The next challenge is collecting data in such a way that you can analyze across very diverse studies, all with their own methods, objectives, and data presentations. Here, I had some questions and suggestions on the classifications used because I'm not sure that all are optimal. The next big challenge is synthesizing the wide range of results into a narrative that can fit into a short format journal. One component of doing so is to create figures that convey results across analyses, which I think the authors did well with their visuals. The other component is writing a single, digestible story while conveying the vast range of information. I think the authors have mostly succeeded at the last part.

Reply: Thank you for your really supportive comments.

Major comments (essential to address):

Results & Discussion
L108-116: there don't seem to be any statistical tests underlying these results. The assessments would be stronger if statistical differences were tested for.

One suggestion would be, for example with the endangered status, the species is either 0 = not threated or 1 = threatened, and analyze the data with a binomial distribution such as: 
Threated (0/1) ~ eats crops (0/1) + (possibly a taxa random effect), (family = binominal) 
Where each row of the data is a species. Or for geographical statements:
Eats crops (0/1) ~ continent + (possibly a taxa random effect), (family = binomial)

Followed by a likelihood ratio test to see if continent matters, then follow up with a Tukey HSD if continent does matter based on the likelihood ratio test.

Reply: Thanks for your suggestions on the statistical tests. We conducted the suggested analysis using the proposed formula. In the model, we assigned the taxonomic ‘family’ as the random effect, the results are printed below (also in the Supplementary HTML file).

This analysis revealed that a crop-consuming bird species, compared to one that does not, is less likely to be endangered. However, we did not include this particular result in the main text because there are many studies working on this question and used more complex models controlling many other confounding factors (e.g, Phylogenetic Generalised Linear Mixed Models), such as “Socioeconomic development and ecological traits as predictors of human-bird conflicts”. That study also found that “species with a relatively greater conflict extent had a relatively broader diet (estimate 0.80 [SE 0.22], p<0.05) and an increasing population trend (estimate 0.58 [SE 0.15], p<0.05)”. As for the geographical statement, we chose to use a gridded richness map to illustrate the spatial and geographical differences. Unfortunately, this test would require a spatial analysis of not only our listed 1157 crop-consuming species, but also the remaining ~9000 species, which is beyond the scope of our paper.

Figure 3: I was quite confused as to why there would be a positive effect for fruit since there's a lot of literature on how fruit farmers think birds cause a lot of damage (e.g., Kross, S. M., Ingram, K. P., Long, R. F., & Niles, M. T. (2018). Farmer perceptions and behaviors related to wildlife and on‐farm conservation actions. Conservation Letters, 11(1), e12364)

. However, later the paper says that coffee was included in fruit and drove the results of finding that birds are beneficial to fruit. I think the results would be more informative if fruit were separated from coffee. For example, the FAO separates fruit from coffee in their classifications: https://www.fao.org/economic/the-statistics-division-ess/methodology/methodology-systems/crops-statistics-concepts-definitions-and-classifications/en/#:~:text=Bananas%2C%20plantains%2C%20grapes%2C%20dates,are%20not%20considered%20fruit%20crops.

Reply: This is an excellent suggestion. In our revision, we have separated studies on “Coffee and cacao” from “Fruit”. And the results have been re-analyzed and re-plotted in Fig 3b (see below).

L226: What is the difference between "birds in general" and "others"? Is "birds in general" studies that you can't get to a species level? Also, why separate crows from magpies and not use general corvids?

Reply: Thanks for the comment and suggestion. Considering the definitions from the original case studies and their sample sizes, we have re-classified the categories: birds in general (if we see the term ‘bird’ in surveys), herbivorous wildfowls (e.g., crane, geese, peafowl, and rhea), corvids (i.e., crows and magpie), or others (e.g., tit, toucan, parrot, dove, blackbird, and hummingbird). We have since updated our analyses and figures.

Methods:
L332-341: My biggest concern is with the study search. It may have followed great protocols, but there is not enough information to assess that as written. What were the search terms used?

Reply: This is also picked up by the rest of the reviewers. We have updated the methods in our revision for the ease of the readers. We actually used the PICO framework (participants, intervention, comparison, and outcome) to search the literature. In our context, we defined the search rules by combinations of animal, experiment/survey method (e.g., exclosure and questionnaire), target (e.g., crop and farmer), and outcome (e.g., production and attitude), such as the combination of ‘bird+exclosure+coffee+yield’ when searching for exclosure experiments.

For the three sub-topics datasets, we initially focused on birds and bats; for the mitigation dataset, we initially evaluate the effectiveness of measures in reducing the loss of crops, fishery, and poultry caused by birds and bats. While the below search keys drew up a longer and broader list of papers, the final meta-analysis was only focused on birds and crops.

**Rules for exclosure experiment dataset**

**Rule1** TS=(bird OR raptor OR "*species name of bird*" OR bat)

**Rule2** TS=(exclud* OR exclosure OR exclusion OR net OR wire OR service OR disservice)

**Rule3** TS=(crop OR agriculture OR corn OR sunflower OR tuber OR seed OR maize OR wheat OR *nut OR cherry OR berry OR rice OR soybean OR sorghum OR fruit OR orchard OR apple OR vegetable)

**Rule4** TS=(yield OR output OR product* OR los* OR biomass OR damage)

**Rule 5 Exclusion** TS=(broiler OR clinical OR muscle OR metabolism OR dairy OR immun* OR cell OR nerve OR genetic OR genomic OR poultry OR egg)

**Rules for mitigation dataset**

**Rule1** TS=(bird OR raptor OR "*species name of bird*" OR bat)

**Rule2** TS=(conflict OR damag* OR feed OR raid OR consum* OR predat* OR kill* OR prey)

**Rule3** TS=(crop OR agriculture OR corn OR sunflower OR tuber OR seed OR maize OR wheat OR *nut OR cherry OR berry OR rice OR soybean OR sorghum OR fruit OR orchard OR apple OR vegetable OR fish* OR pet OR livestock OR chick* OR game* OR peasant OR poult* OR grouse OR goat OR cow OR lamb)

**Rule4** TS=(yield OR output OR product* OR los* OR death OR biomass OR mortality OR stock OR attitude OR perception)

**Rule5** TS=(protect* OR manag* OR *control OR mitigation OR mitgat* OR repel* OR compensat* OR guard* OR trap OR scar* OR bait* OR cull* OR avers* OR fenc* OR poison* OR shoot*)

**Rule6 Exclusion** TS=(broiler OR clinical OR muscle OR metabolism OR dairy OR immun* OR cell OR nerve OR genetic OR genomic OR poultry OR egg)

**Rules for attitude dataset**

**Rule1** TS=(bird OR raptor OR "*species name of bird*" OR bat)

**Rule2** TS=(farm* OR village* OR suburb OR countryside OR community OR coexist* OR “nature reserve” OR “national park” OR livelihood OR hunter OR public OR crop OR agriculture OR fruit OR orchard OR vegetable OR citizen OR tourist OR visitor OR urban OR city)

**Rule3** TS=(attitude OR perception OR awareness OR belief)

Please refer to more details under the ‘Search rules’ in Supplemental Information.

Some studies seem to have been missed by the protocol used. For example, one study that is missing in the social side that seemingly fits the criteria is Kross, S. M., Ingram, K. P., Long, R. F., & Niles, M. T. (2018). Farmer perceptions and behaviors related to wildlife and on‐farm conservation actions. Conservation Letters, 11(1), e12364.

Reply: Thanks for highlighting this reference. However, Kross et al. reported the results in a Likert scale (please refer to the extracted the plot below), as such the proportion data is not particularly suited for our study.

Minor comments (smaller suggestions for improvement):
Title: The title made me think that you were reviewing prior meta-analyses rather than conducting a series of analyses in your meta-analysis. I think the first part of the title is good but the part after the colon should be changed.

Reply: Good point. We have changed the title to “Coexisting with birds in cropland: Meta-analyses of birds ex-closure and crop loss mitigation experiments, and insights from social surveys”

Introduction
L59-60: need reference

Reply: We have included some references.

L60-63: need reference

Reply: We have added some references.

L71: I think "direct (seen) damage to crops" would fit better here

Reply: Thanks. We have edited the sentence.

L79: Is it "public perception" that you're looing at or specifically "producers' perceptions"?

Reply: We are looking at public perception including those from farmers.

Results & Discussion:
L103 and throughout: I am not sure if "Handbook of the" is needed rather than just "Birds of the World." I thought that it might have been a different source than the Cornell Lab of Ornithology's Birds of the World until I clicked the link in the Methods.

Reply: It is the ‘Birds of the World’, which was data amassed from ‘Birds of North America’, ‘The Handbook of Birds of the World’, ‘Neotropical Birds’, and ‘Bird Families of the World’. And it is by far the most comprehensive document about bird life history at the global scale.

L129-130: The use of bird access as experimental treatments seems strange to me since that's the condition in absence of treatments (which is generally called the control).

Reply: Thanks for your suggestion here. Yes, it does seem a little strange, but it is more convenient and intuitive when interpreting our results. That is, if the effect size is positive, we can say the birds have a positive effect on crops.

L159-162: Looking at the reference, the total applied toxicity per substances has decreased for birds over time, but it is not zero. You could say “…because the total applied toxicity per substance has decreased for birds over the last couple decades [22]”

Reply: Thank you for picking this up. We have deleted this statement in the revision.

L182: I was confused where 114 experiments came from, looking at the number of studies. I think “…114 effect sizes from the agricultural fields…” would be more accurate

Reply: Thanks for your advice. We have revised the manuscript accordingly.

L189: I think maybe "all-stages" works better? I think you're saying that some measures are just for the sowing period but others go across all periods?

Reply: Thanks. We have revised the manuscript accordingly.

L190: I think "However, we…" might work better than "Therefore, we…"

Reply: We have rephrased these two sentences in the main text.

L227: "CGPR" was not intuitive to me, so I forgot what this was referring to when it came up again. Maybe "conflict-prone" or something similar would be better to use after the first mention if you need something shorter than "conflict-prone large birds"

Reply: Good suggestion. We have used “herbivorous wildfowl” in the revision.

L259-262: Did you find any evidence in the social literature that these could be effective that you could use as a reference? There are agencies and nonprofits doing this, but my understanding is that farmer uptake of bird friendly practices is still low.

Reply: Yes, we have added the following three references to this statement.

Piñeiro V, Arias J, Dürr J, Elverdin P, Ibáñez AM, Kinengyere A, et al. A scoping review on incentives for adoption of sustainable agricultural practices and their outcomes. Nature Sustainability. 2020;3(10):809-20. doi: 10.1038/s41893-020-00617-y.

Mishra C, Allen P, McCarthy T, Madhusudan M, Bayarjargal A, Prins HH. The role of incentive programs in conserving the snow leopard. Conservation Biology. 2003;17(6):1512-20.

Basnet D, Jianmei Y, Dorji T, Qianli X, Lama AK, Maowei Y, et al. Bird photography tourism, sustainable livelihoods, and biodiversity conservation: a case study from China. Mountain Research and Development. 2021;41(2):D1.

Methods
L325: The right citation for the Birds of the World it is at https://birdsoftheworld.org/bow/support/citations-and-references 
"To cite Birds of the World as a whole (for example, you want to refer to the series in general or you have used content from many accounts):
Birds of the World (2022). Edited by S. M. Billerman, B. K. Keeney, P. G. Rodewald, and T. S. Schulenberg. Cornell Laboratory of Ornithology, Ithaca, NY, USA. https://birdsoftheworld.org/bow/home
…or, if you need to go with a more standard author/date format:
S. M. Billerman, B. K. Keeney, P. G. Rodewald, and T. S. Schulenberg (Editors) (2022). Birds of the World. Cornell Laboratory of Ornithology, Ithaca, NY, USA. <https://birdsoftheworld.org/bow/home>"

Reply: Thanks for your suggestion. We are using “Billerman S, Keeney B, Rodewald P, Schulenberg T. Birds of the World. Cornell Laboratory of Ornithology, Ithaca, NY, USA. 2020.”

L333: add last month searched in 2020

Reply: We added this description in the Supplementary Information.

L351: is the list an "i.e.," = "that is" or an "e.g.," = "for example." If "i.e.," then it's indicating that these were the only birds but "e.g.," would be saying that these are some examples

Reply: Correct, we used these two words in the same way.

L361-366: How did you determine a location to extract data from and what is the spatial resolution of this map? In my experience, most papers will not have very exact sampling locations that can be used for precise landscape data extraction.

Reply: We compiled the location information from the author’s reported latitudes and longitudes or the approximate centroid of study sites from the case studies. We have now added this description in the main text.

When you say "landscape" what scale do you actually mean? I don't think you necessarily need to change your methods, but I don't think "landscape-level bird richness" is probably the right description. Perhaps "regional bird richness" is correct, depending on your scale (terms also used at L159).

Reply: Thanks for your suggestion, we have used “regional bird richness” in our revision.

Supporting Materials:
Table S2: Raspberry is missing a p, and I think "sour cherry" should be two words (but then is the "cherry" above just sweet cherry or binned sweet/sour?)

Reply: Thanks. We have corrected the spelling. And we have now binned these two together, so the total number of crop types is 88, instead of 89.

Table S3: I don't think the main text says that "metafor" was used specifically.

Reply: We have added the citation of the “metafor” package in the main text.
