# Supplementary material for: Insights into the coexistence of birds and humans in cropland through meta-analyses of bird exclosure studies, crop loss mitigation experiments, and social surveys
Source: PLoS Biol. 2023 Jul 6;21(7):e3002166. doi: 10.1371/journal.pbio.3002166 (PMC10325107; doi:10.1371/journal.pbio.3002166)
Supplement: Attachment — Submitted filename: Response to Editor.docx [file pbio.3002166.s010.docx]

Dear Dr Lee, 

Thank you for submitting your manuscript "Coexisting with birds in cropland: Meta-analyses of birds exclosure and crop loss mitigation experiments, and insights from social surveys" to PLOS Biology. We are looking forward to passing your manuscript on for evaluation; however, after an initial check of your files, we would first ask you to address the following points:       


1.    Please upload all main figures as separate Figure files in .tif or .eps format. For more information about how to convert and format your figure files please see our guidelines: https://journals.plos.org/plosbiology/s/figures

Reply: we uploaded figures separately.

2.    We have noticed that you have uploaded Supporting Information files, but you have not included a list of detailed legends. Please add a full list of legends for your Supporting Information files after the references list (including legends for your Supplementary Data files).

Reply: we added full legends for six datasets, twelve tables, and eight figures.

3.    Please ensure that the funders and grant numbers match between the Financial Disclosure field and the Funding Information tab in your submission form. Note that the funders must be provided in the same order in both places as well.

-  State what role the funders took in the study. If the funders had no role in your study, please state: “The funders had no role in study design, data collection and analysis, decision to publish, or preparation of the manuscript.” 

4.    Some material included in your submission may be copyrighted. According to PLOS’s copyright policy, authors who use figures or other material (e.g., graphics, clipart, maps) from another author or copyright holder must demonstrate or obtain permission to publish this material under the Creative Commons Attribution 4.0 International (CC BY 4.0) License used by PLOS journals. Please closely review the details of PLOS’s copyright requirements here: PLOS Licenses and Copyright. If you need to request permissions from a copyright holder, you may use PLOS's Copyright Content Permission form. 

Please respond directly to this email and provide any known details concerning your material's license terms and permissions required for reuse, even if you have not yet obtained copyright permissions or are unsure of your material's copyright compatibility. Once you have responded and addressed all other outstanding technical requirements, you may resubmit your manuscript within Editorial Manager. 

Potential Copyright Issues: 
-    Fig 1, Fig 5: Please confirm whether you drew the images / clip-art within the figure panels by hand. If you did not draw the images, please provide (a) a link to the source of the images or icons and their license / terms of use; or (b) written permission from the copyright holder to publish the images or icons under our CC BY 4.0 license. Alternatively, you may replace the images with open source alternatives. See these open source resources you may use to replace images / clip-art: https://commons.wikimedia.org; https://openclipart.org/.

Reply:

Clip-arts of birds in Fig 1a were from <https://www.phylopic.org/>.

Clip-arts of the key and farmer in Fig 1a were from <https://openclipart.org/>.

Clip-arts of birds and insects in Fig 1c were from <https://www.phylopic.org/>.

We draw other images and clip-arts.

-    Fig 2; Fig 7; S3 Fig; S6 Fig; S7 Fig; S8 Fig : please (a) provide a direct link to the base layer of the map (i.e., the country or region border shape) and ensure this is also included in the figure legend; and (b) provide a link to the terms of use / license information for the base layer image or shapefile. We cannot publish proprietary or copyrighted maps (e.g. Google Maps, Mapquest) and the terms of use for your map base layer must be compatible with our CC BY 4.0 license. 

Reply: The base map of country boundaries was from https://www.naturalearthdata.com/. We added the link in figure legend.

-    Note: if you created the map in a software program like R or ArcGIS, please locate and indicate the source of the basemap shapefile onto which data has been plotted. 

-    If your map was obtained from a copyrighted source please amend the figure so that the base map used is from an openly available source. Alternatively, please provide explicit written permission from the copyright holder granting you the right to publish the material under our CC BY 4.0 license. 

-    If you are unsure whether you can use a map or not, please do reach out and we will be able to help you. The following websites are good examples of where you can source open access or public domain maps: 
* U.S. Geological Survey (USGS) - All maps are in the public domain. (http://www.usgs.gov) 
* PlaniGlobe - All maps are published under a Creative Commons license so please cite “PlaniGlobe, http://www.planiglobe.com, CC BY 2.0” in the image credit after the caption. (http://www.planiglobe.com/?lang=enl) 
* Natural Earth - All maps are public domain. (http://www.naturalearthdata.com/about/terms-of-use/) 


You will find your submission in the 'Submissions Sent Back to Author' folder. Once you have made these changes, please re-approve the submission following the same steps as before.      

Please do not update your Response to Reviewers file with the changes made as a result of these requests.     

Please resolve these issues and resubmit your manuscript within 30 days. If you need more time, please email the journal office at [plosbiology@plos.org](mailto:plosbiology@plos.org). We are happy to grant extensions of up to one month past this due date. If we do not hear from you within 30 days, we will withdraw your manuscript on your behalf. 

If you have any questions or require assistance, please don't hesitate to get in touch. To help us minimize inbox volumes, we kindly ask that you not reply to this email simply to confirm you have resubmitted your revisions. 

Kind regards,          

Livia Horvath 
Publications Assistant 
PLOS Biology
