# Supplementary material for: Insights into the coexistence of birds and humans in cropland through meta-analyses of bird exclosure studies, crop loss mitigation experiments, and social surveys
Source: PLoS Biol. 2023 Jul 6;21(7):e3002166. doi: 10.1371/journal.pbio.3002166 (PMC10325107; doi:10.1371/journal.pbio.3002166)
Supplement: Attachment — Submitted filename: Response to Reviewers.docx [file pbio.3002166.s011.docx]

We thank the reviewer for his/her efforts to improve our manuscript. We have revised our manuscript accordingly.

Reviewer #1:
I found that the authors have done an excellent job for responding to most of my comments. I especially like the newly added analysis of people's attitudes, which I believe has made the paper's message much more robust.

Reply: Thank you for your endorsement.

I now have only a few minor comments on Supplementary Information.

1. P2, L14: Replace participation with population and comparison with comparator.

Reply: We have revised the manuscript accordingly.

2. P2, L22-24: I suggested giving the full detail of how non-English literature was searched, such as databases used, search terms, target languages etc, but the authors only provided the following explanation, which I don't think is sufficient. Please provide the full details.

"In Oct 2022, using similar approaches above, we re-searched non-English literature published before December 2020 and added only one study in Spanish in the exclosure experiment dataset."

Reply: We appreciate this suggestion. We have now clarified the conduct of non-English literature search in the Supplementary Information. It reads:

“*In Oct 2022, using the same databases in the Web of Science and search terms as the initial search above, we re-searched non-English literature published before December 2020 and added only one study in Spanish in the exclosure experiment dataset*.”
